# A genomic and evolutionary approach reveals non-genetic drug resistance in malaria

**DOI:** 10.1186/s13059-014-0511-2

**Published:** 2014-11-14

**Authors:** Jonathan D Herman, Daniel P Rice, Ulf Ribacke, Jacob Silterra, Amy A Deik, Eli L Moss, Kate M Broadbent, Daniel E Neafsey, Michael M Desai, Clary B Clish, Ralph Mazitschek, Dyann F Wirth

**Affiliations:** Department of Immunology and Infectious Disease, Harvard School of Public Health, Boston, MA 02115 USA; Broad Institute of MIT and Harvard, Cambridge, MA 02142 USA; Biological and Biomedical Sciences, Boston, MA 02115 USA; Harvard/MIT Division of Health Sciences and Technology, Boston, MA 02115 USA; Harvard/MIT MD-PhD Program, Harvard Medical School, Boston, MA 02115 USA; Department of Organismic and Evolutionary Biology, Harvard University, Cambridge, MA 02138 USA; FAS Center for Systems Biology, Harvard University, Cambridge, MA 02138 USA; Department of Cell and Molecular Biology, Microbiology, Uppsala University, Uppsala, 75105 Sweden; Massachusetts General Hospital, Center for Systems Biology, Boston, MA 02114 USA

## Abstract

**Background:**

Drug resistance remains a major public health challenge for malaria treatment and eradication. Individual loci associated with drug resistance to many antimalarials have been identified, but their epistasis with other resistance mechanisms has not yet been elucidated.

**Results:**

We previously described two mutations in the cytoplasmic prolyl-tRNA synthetase (cPRS) gene that confer resistance to halofuginone. We describe here the evolutionary trajectory of halofuginone resistance of two independent drug resistance selections in *Plasmodium falciparum*. Using this novel methodology, we discover an unexpected non-genetic drug resistance mechanism that *P. falciparum* utilizes before genetic modification of the cPRS*. P. falciparum* first upregulates its proline amino acid homeostasis in response to halofuginone pressure. We show that this non-genetic adaptation to halofuginone is not likely mediated by differential RNA expression and precedes mutation or amplification of the *cPRS* gene. By tracking the evolution of the two drug resistance selections with whole genome sequencing, we further demonstrate that the *cPRS* locus accounts for the majority of genetic adaptation to halofuginone in *P. falciparum*. We further validate that copy-number variations at the *cPRS* locus also contribute to halofuginone resistance.

**Conclusions:**

We provide a three-step model for multi-locus evolution of halofuginone drug resistance in *P. falciparum*. Informed by genomic approaches, our results provide the first comprehensive view of the evolutionary trajectory malaria parasites take to achieve drug resistance. Our understanding of the multiple genetic and non-genetic mechanisms of drug resistance informs how we will design and pair future anti-malarials for clinical use.

**Electronic supplementary material:**

The online version of this article (doi:10.1186/s13059-014-0511-2) contains supplementary material, which is available to authorized users.

## Background

Malaria is a treatable communicable disease yet remains a common cause of death and disease especially among pregnant women and children. Most of malaria’s worldwide burden disproportionately lies in Southeast Asia and Sub-Saharan Africa, causing over 1.2 million deaths in 2010 [1]. Western medicine’s 100+ year history of combating *Plasmodium falciparum* has taught us that the global population of malaria parasites has a unique and dangerous ability to rapidly evolve and spread drug resistance. Recently it was documented that resistance to the first-line antimalarial artemisinin may be developing in Southeast Asia and the molecular underpinnings of artemisinin delayed clearance are begging to be characterized [2,3].

Consequently, it is essential to find new families of antimalarial molecules to take over if artemisinin and its combination-based therapies continue to lose efficacy. Forward genetic drug resistance screening and genomic analysis have previously been used to identify new targets for drug development and understand new drug resistance mechanisms [4–9]. The targets of more than 12 families of small molecules (reviewed in [10]) have been identified in *Plasmodia* through *in vitro* selection and genomic characterization of the end-points of these selections. This approach inherently assumes a single mechanism of drug resistance and overlooks the temporality of genetic and non-genetic epistasis involved in the complex evolution of drug resistance in a eukaryotic parasite with a genome of approximately 23 megabases and roughly 5,500 expressed proteins in the parasites’ life cycles [11].

The dynamics of evolution are essential to understand the drug resistance phenotypes that are readily achieved by Darwinian evolution. Studies in bacterial drug resistance have shown that limited pathways are available due to epistatic interactions between *cis*- and *trans*-interacting genetic alterations [12]. Epistasis among genes strongly shape the evolution of microbes [13–15] and viruses [16–18]. Whole-genome whole-population sequencing in bacterial [19] and yeast [20,21] laboratory evolution experiments have proven informative of the evolutionary dynamics at play in long-term adaptation to a variety of selective pressures.

We chose to use a whole-genome whole-population sequencing approach to track the evolutionary dynamics of resistance to the *P. falciparum cytoplasmic proline tRNA synthetase* (*cPRS*) inhibitor halofuginone. tRNA synthetases are very promising anti-parasitic targets for the development novel antimalarials [22] and recent work has found small molecule antimalarials that target the isoleucine tRNA synthetase [23], lysine tRNA synthetase [7], threonine tRNA synthetase [24], and our own work on proline tRNA synthetase (JD Herman *et al.*, submitted). Understanding this interplay of multiple resistance mechanisms is essential for target prioritization, combination therapy design, and drug-resistance surveillance.

## Results

### Mutations in the *cPRS* gene cannot explain evolution of resistance in all long-term selected lines

We previously identified the *cPRS* gene as the molecular target of halofuginone, and related small molecules (JD Herman *et al.*, submitted). We discovered two unique non-synonymous mutations in the *cPRS* gene in independent end-point *in vitro* selection experiments. In this work, we observed that the parasite population gradually acquired resistance to increasing concentrations of halofuginone during the *in vitro* selection process and we sought to understand this evolution of resistance at a molecular level. Using recent advances in genome sequencing technology and new analytical methods, we characterized two independent selections along their *in vitro* evolutionary trajectory.

**Figure 1:**
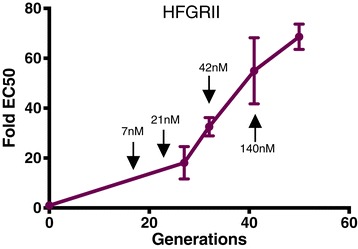
**HFGRII immediately acquires drug resistance during long-term halofuginone selection.** HFGRII was drug phenotyped at 27, 34, 41, and 50 generations along the selection. The black arrows identify when the corresponding halofuginone drug concentration was increased to 7, 21, 42, and 140 nM. Resistance is quantified as the fold-increase in EC50 of the total population over the EC50 of the Dd2 parental line. EC50s were determined by a tritiated hypoxanthine incorporation dose-response assay. Error bars denote standard deviation.

The *P. falciparum* Dd2 lines halofuginone resistance selected line II (HFGRII) and halofuginone resistance selected line II (HFGRIII) were selected in parallel with an intermittent stepwise strong-selective pressure protocol. Selections began with 10× the parental EC50 for halofuginone (7 nM) and were increased stepwise upon growth tolerance (Additional files 1, 2, and 3). Both HFGRII and HFGRIII grew tolerant of 7 nM halofuginone in 18 generations, 21 nM in 9 generations, 42 nM in 7 and 9 generations, respectively, and 140 nM in 16 and 22.5 generations, respectively. To confirm these phenotypes, we measured halofuginone dose-response of HFGRII in standard growth assays at selected time points (Figure 1). Consistent with the bulk population growth, HFGRII displayed a constant response to increasing selective pressure.

### Genomic mutations only appear after the onset of drug resistance

We performed whole genome sequencing of the entire evolving population to track the rise and fall of mutant alleles over 50 generations (HFGRII) and 58.5 generations (HFGRIII) (displayed in Additional files 1 and 2). We used our time-course data from HFGRII and HFGRIII to distinguish true mutations from sequencing or alignment-introduced error (filtering scheme described in Additional file 4). Since we were interested in *de novo* mutational adaptation and our selections began with clonal strains, the two independent replicate populations should not share the exact same mutations. Second, the frequencies of a real mutation should correlate through time (positive autocorrelation), while sequencing errors should be uncorrelated at different time points (negative or zero autocorrelation). We used both *de novo* mutation and autocorrelation to identify the segregating SNPs and small indels in the independent populations.

**Figure 2:**
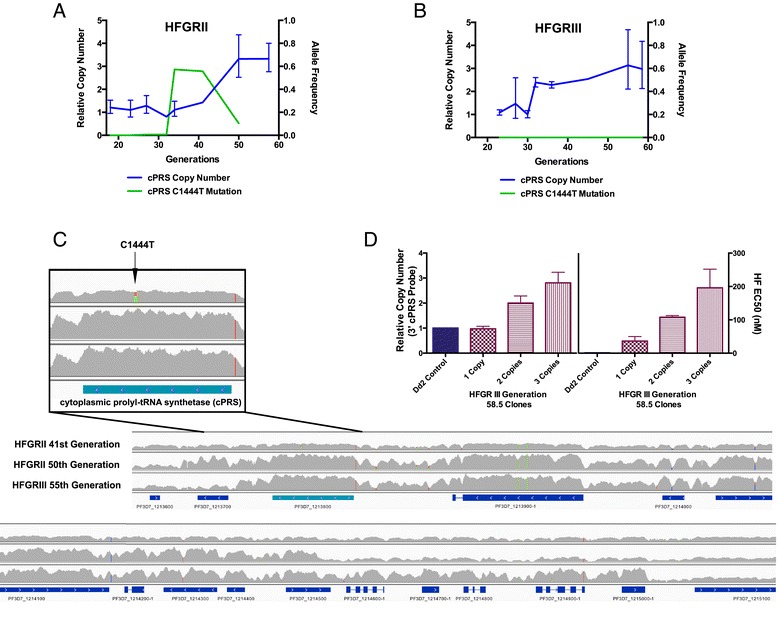
**Genetic adaption at the** ***cPRS*** **locus accounts for acquisition of halofuginone resistance after generation 32 in HFGRII and HFGRIII. (A,B)** Quantitative PCR copy number and allele type of uncloned HFGRII (A) and HFGRIII (B) revealed parasites with mutant *cPRS* alleles that failed to proceed to fixation in either population in favor of clones with wild-type amplified loci. In HFGRII, mutant parasite clones reached a maximum allele frequency of 0.57 and were competed out by those with amplified wild-type loci. In HFGRIII, parasite clones with mutant *cPRS* loci were undetectable. Neither *cPRS* mutation nor amplification reached sufficient allele frequency before the 34th (HFGRII) or 32nd (HFGRIII) generation after selection with 42 nM halofuginone (60× EC50). **(C)** Though HFGRII and HFGRIII have different amplification breakpoints as illustrated by the next-generation sequencing read pileups, both include wild-type *cPRS* (PF3D7_1213800) alleles. The HFGRII 41st generation pileup confirms that the *cPRS* locus is unamplified and reflects a mixture of wild-type and mutant haploid parasites. **(D)** The natural allelic series of HFGRIII generation 58.5 clones with 1, 2, and 3 copies confirms that amplification of the *cPRS* locus confers resistance to halofuginone. Copy number variation determined by quantitative PCR of three clones investigated for sensitivity to halofuginone using the 3′ cPRS assay. SYBR growth dose-response assay confirms that more cPRS copies desensitize parasites to halofuginone. Relative copy number in (A,B,D) was determined with SerRS as an endogenous control to a single *cPRS* copy Dd2 parasite. Allele frequencies were determined from whole-genome sequencing. Read pileups in (C) were generated from aligned reads generated on an Illuminia HiSeq 2000 and visualized with IGV v 2.3.32. Error bars in (D) denote standard deviation.

From this analysis framework, we found a paucity of genomic mutations over time (Additional file 5). Most of the genetic changes during our evolution experiments occurred at the *cPRS* locus. With this method, we confirmed the evolution of the C1444T (L482F) *cPRS* mutation in HFGRII. The C1444T mutation first appeared in the 27th generation at a 0.6% allele frequency (Figure 2A). However, the *cPRS* mutation in HFGRII did not proceed to fixation. It reached a maximum allele frequency of 57%, then after 20 additional generations fell from the population.

To supplement the Genome Analysis Toolkit (GATK)-based analysis of small indels, we searched the pileups from whole-genome sequenced populations for long-scale amplification. We found that the *cPRS* was part of larger amplifications at later time points in HFGRII and HFGRIII (Figure 2A-C). To better quantify the amplification of *cPRS* over time in both selections, we performed relative quantification of HFGRII (Figure 2A) and HFGRIII (Figure 2B) using quantitative PCR (qPCR). We found that the *cPRS* copy number of HFGRII rose as the C1444T *cPRS* mutation fell in the population. The strong anticorrelation of the two segregating sites implies clonal competition.

Using the same methodologies, we found that no SNPs proceeded to fixation in HFGRIII over the course of the experiment (Figure 2B). Rather, in the 32nd generation of the HFGRIII selection, parasites with wild-type (WT) amplified loci first appeared and remained for the duration of the selection. However, from these data we can only determine the average number of *cPRS* copies across the entire population. To determine the population distribution of parasites with WT amplified *cPRS* loci, we performed dilutional cloning on the 55th generation of HFGRIII. From 14 cloned parasites, we determined that 71% of the HFGRIII 55th generation had three WT copies and 21% had two WT copies of the *cPRS* locus (Additional file 6). To explore the effect that *cPRS* copy number variation plays in resistance to halofuginone, we choose three parasite clones with 1, 2, or 3 copies of *cPRS* (Figure 2D). We found that halofuginone resistance increased with the number of copies of *cPRS* (Figure 2D) and there was no change in the EC50 of 10 other antimalarial compounds we tested (Additional file 7).

From our whole-genome analysis, we identified genetic adaptations at the *cPRS* locus from generation 32 and beyond, in both *in vitro* evolution experiments. However, acquisition of halofuginone resistance preceded *cPRS* amplification or mutations (Figure 1). By cycle 27 in HFGRII, the bulk population had 18-fold decreased sensitivity to halofuginone (Figure 1) that we cannot explain from our genetic analysis. Without mutations sweeping to fixation, early drug resistance might be achieved in these populations by multiple mutations in independent lineages and would have been missed by metagenomic sequencing. However, the conservative mutation rate and few accumulated mutations observed in a similar *P. falciparum in vitro* culture system [25] argue that a non-genetic adaptive mechanism is more likely.

### Induced halofuginone resistance - a stable resistance acquired in eight generations

**Figure 3:**
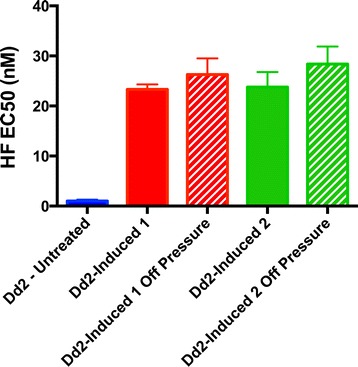
**Induction of halofuginone resistance occurs in eight generations and is stable.** After eight generations of exposure to 4× EC50 of halofuginone (2.8 nM), both Dd2 replicates, Dd2 Induced 1 and 2, recrudesced and became 20- to 30-fold resistant to halofuginone treatment. This phenotype was stable during freeze-thaws and after 30+ generations of growth of Dd2 Induced 1 and 2 in the absence of halofuginone (Dd2-Induced 1 Off Pressure and Dd2-Induced 2 Off Pressure). EC50s were determined by SYBR green growth assay. Error bars denote standard deviation.

We sought to understand the early phase of acquired resistance that was independent of any genetic element we could identify. To probe the early genetic or non-genetic mechanisms of halofuginone resistance, we created the halofuginone-induced resistance model. We put clonal WT Dd2 parasites under constant low halofuginone pressure (2.8 nM) five-fold lower than that used in the first step of the selection of HFGRII and HFGRIII. At first, the parasite populations decreased below the detection limit of thin smear microscopy. Within eight to nine generations, both replicate-treated Dd2 lines recrudesced and were more than 20-fold resistant compared with an untreated Dd2 line (Figure 3). This induced resistance was stable and heritable in both Dd2 biological replicates, Dd2 Induced 1 and Dd2 Induced 2, for greater than 30 generations without drug pressure. We also replicated these experiments in the unrelated parasite line HB3 (Additional file 8). First we validated that Dd2 Induced 1 and 2 had no mutations or copy number variations at the *cPRS* locus (Additional file 9). Next, we performed whole-genome sequencing on the parental strain and Dd2 Induced 1 and 2. Of the five genes with non-synonymous SNPs called between the parental and induced strains, four were eliminated as alignment/SNP-calling error with Sanger sequencing (Additional file 10) and a fifth was not found in replicate inductions. Thus, we concluded sweeping genetic adaptation could not account for the halofuginone induction phenotype.

### Increased proline concentration results in abrupt induction of halofuginone resistance

We wanted to understand the mechanism of induced resistance and therefore investigated amino acid homeostasis in our induced parasites. Proline is the only of cPRS’s three substrates (ATP, proline, and uncharged proline tRNA) that we believe competes with halofuginone for the cPRS binding pocket based on structural studies (JD Herman *et al.*, submitted) [26,27]. We investigated whether parasites with halofuginone-induced resistance have altered amino acid homeostasis using a liquid crystallography-mass spectrometry (LC-MS) metabolomics approach.

**Figure 4:**
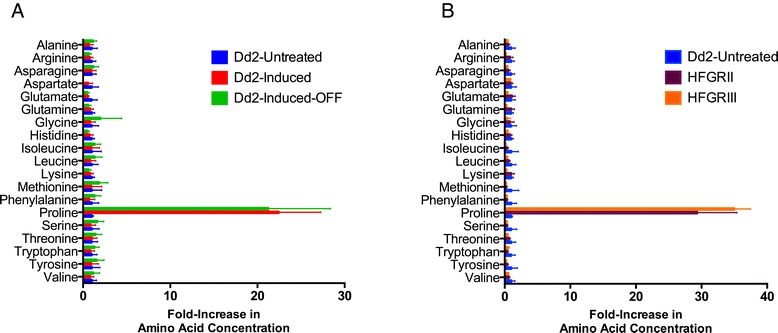
**Halofuginone short-term induced and long-term selected parasites specifically upregulate intracellular proline concentration. (A**,**B)** Of the 19 proteogenic amino acids assayed from saponin-released purified parasites, only proline concentrations were increased in Dd2 Induced parasites (A) and long-term selected lines HFGRII and HFGRIII (B). The fold increase in amino acid concentration upon induction or selection is expressed as a ratio of induced/selected line measurement over parental Dd2 line measurement. Amino acid levels of saponin-released *P. falciparum* parasites were quantified from the normalized integrated peak intensity determined by LC-MS. Error bars denote standard deviation.

We measured 19 intracellular amino acid levels in our Dd2 Induced 1 and Dd2 Induced 2 parasites and found proline to be uniquely upregulated. We examined the metabolomics picture of these parasites by measuring both saponin-freed *P. falciparum* (Figure 4) and *P. falciparum* infected red blood cells (iRBCs) (Figure S4a in Additional file 11). This provided further insight into the dynamics of proline accumulation that may contribute to induced halofuginone resistance. Both free parasites and total iRBCs had increased proline concentrations when compared with the parental Dd2 line (Figure 4A; Additional file 11). The free parasite fraction had a 19- to 32-fold increase while the total iRBC sample had a 3- to 5-fold increase, consistent with the parasite cytosol as the source of metabolic enrichment. To validate that this is a cell line autonomous phenomenon, we tested and found proline-specific upregulation in halofuginone-induced HB3 parasites as well (Figure S5b,c in Additional file 8).

### Increases in proline are strongly associated with halofuginone resistance

To understand the initial timing and subsequent dynamics of induced halofuginone resistance associated with increased proline, we measured intracellular proline concentration immediately after recrudescence and in subsequent generations of Dd2 Induced parasites. We found that proline concentrations were increased upon our first technically feasible measurement and did not vary significantly up to 60 generations after, while parasites were maintained under constant halofuginone pressure (Figure S6a in Additional file 12).

Further, we wanted to understand if the dynamics of increased proline in the Dd2 Induced parasites corresponded with increased halofuginone resistance. We found that the increase in the halofuginone dose-response was seen as early as the fifth generation post-recrudescence. While maintained on 4× EC50 halofuginone pressure, halofuginone resistance in Dd2 Induced parasites remained constant over 60 generations (Figure S6b in Additional file 12) as did proline levels (Figure S6a in Additional file 12).

Further, we wanted to understand if increased proline autonomous of any cell-intrinsic adaptation could change the dose-response of halofuginone. To this end, we measured the dose-response of halofuginone in untreated Dd2 parasites growing in media with a gradient of proline concentrations in a 72-hour assay. We found that increasing proline concentrations in the growth media can give *P. falciparum* an apparent resistance to halofuginone (Additional file 13) without any pre-conditioning.

### New proline homeostatic set-point is stable

Next we wanted to determine the relevance and permanence of elevated intracellular proline as a resistance mechanism in *P. falciparum.* To test the stability of this proline enrichment, we took Dd2 Induced parasites grown without halofuginone pressure for more than 30 generations (Figure 3) and measured their amino acid levels. We found that both biological replicate Dd2 inductions maintained their drug resistance and their specific upregulation of proline (Figure 4).

To understand whether increased intracellular proline is a shared mechanism of resistance between our short-term induced and long-term halofuginone selected parasites, we measured the amino acid levels of HFGRII and HFGRIII parasites. We found that long-term selected parasites have uniquely elevated proline similar to halofuginone-induced parasites (Figure 4B; Figure S4b in Additional file 11). This further confirmed the stability of the proline-related upregulation and showed that two long-term selected lines, HFGRII and HFGRIII, also use this halofuginone drug-resistance mechanism.

### Proline-like non-proteogenic amino acids are also upregulated during halofuginone-induced resistance

**Figure 5:**
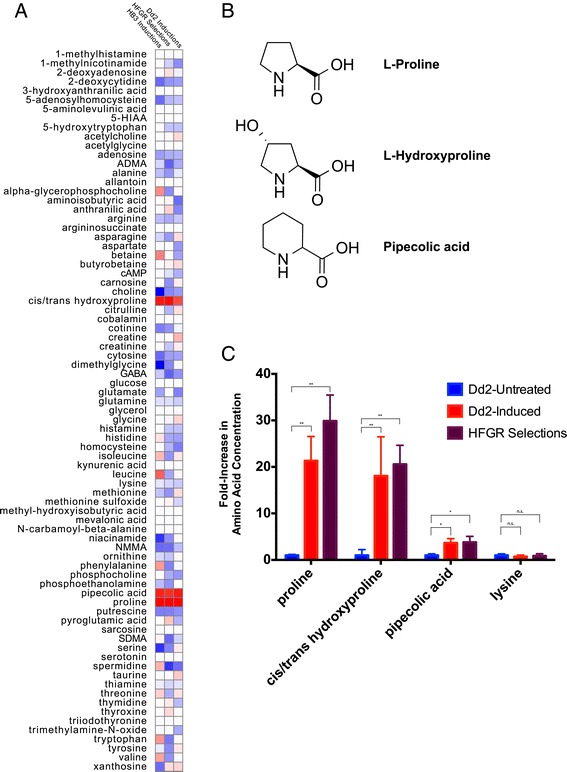
**Profiling of 115 polar metabolites reveals that proline and proline-like non-proteogenic amino acids are uniquely enriched in halofuginone short-term induced and long-term selected parasites. (A)** Of the 115 polar metabolites measured, only pipecolic acid and cis/trans-hydroxyproline concentrations are highly correlated with cytosolic proline concentrations in short-term induced Dd2 parasites, short-term induced HB3 parasites, and long-term selected Dd2 lines HFGRI, II, and III. Each column represents an independent dataset analyzed: HB3 untreated and induced parasites; Dd2 untreated and HFGRII and HFGRIII parasites; and also Dd2 untreated and induced parasites. The full metabolite profile is provided in Additional file 14. **(B)** The three amino acids all contain cyclic secondary amines and carboxylate groups. The addition of a hydroxyl and replacement of a 5-member for a 6-cyclic amine result in the slightly different chemical and physical properties between proline and hydroxyproline and pipecolic acid, respectively. **(C)** Cytosolic proline, cis/trans-hydroxyproline, and pipecolic acid are elevated in halofuginone-resistant parasites compared with an untreated Dd2 line. The cytosolic concentration of the pipecolic acid precursor lysine is unaffected. The heat map in (A) represents the correlation coefficients (r-values) between cytosolic concentrations of metabolites with that of proline in three collections of WT and modified parasites. High correlation (r = 1) is represented by red while low correlation (r = -1) is represented by blue. Statistical significance in (C) was determined with multiple unpaired *t*-tests using the Sidak-Bonferroni method, with alpha = 0.05. Significant results are indicated by asterisks: single asterisks indicate uncorrected *P* < 10^-7^ and double asterisks indicates uncorrected *P* < 10^-10^. Error bars denote standard deviation.

To better understand the specificity of amino acid re-regulation halofuginone induces, we examined the concentration of 96 additional polar metabolites (Additional file 14) in halofuginone short-term induced and long-term selected parasites. We found two metabolites whose parasite intracellular concentration was highly correlated with proline: cis/trans-hydroxyproline and pipecolic acid (homoproline) (Figure 5A,B). These strong correlations were present when limiting analysis to HB3 untreated and induced parasites, Dd2 untreated and HFGRII and HFGRIII parasites, and also Dd2 untreated and induced parasites (Figure 5A). Pipecolic acid, a proline-like non-proteogenic amino acid, is known to be formed by the degradation of lysine and not proline [28]. We also found that hydroxyproline and pipecolic acid were similarly enriched in all parasites with elevated intracellular proline while lysine levels were unchanged (Figure 5C). Though non-enzymatic oxidation of proline could produce hydroxyproline that would be highly correlated with proline levels, pipecolic acid could not be derived from proline non-enzymatically. Additionally, levels of lysine, the metabolic precursor of pipecolic acid, are unperturbed in halofuginone-induced parasites, suggesting a primary enrichment of pipecolic itself (Figure 5C).

### Halofuginone-induced resistance is not associated with changes in gene expression

The enrichment of proline and proline-like amino acids directed our interest to *clag 2* and *3.2* - two members of a multigenic family implicated in the increased flux of proline across the iRBC membrane via the plasmodium-specific anion current [29–32]. After the publication of two papers supporting the involvement of clag genes in the acquisition of resistance to the antimalarial blasticidin [33,34], we became interested in whether a similar epigenetic mechanism explained the rapid acquisition of halofuginone resistance. We found that *clag 2* and *3.2* were not consistently differentially expressed in halofuginone-induced parasites (Figure S9a in Additional file 15). Their pattern of expression varied most consistently based on their maturity within the early schizont stage (approximately 34 hours post-infection (hpi)) rather than between untreated and halofuginone-induced parasites.

We also took a genome-wide approach to evaluate changes in gene expression that could explain the non-genetic resistance to halofuginone and would lead to an epigenetic mechanism. The clag family of genes is one of the few examples of *P. falciparum* genes that show steady-state differences in RNA transcript levels in response to external stimuli [33,34]. Steady-state RNA transcript abundance is hard wired to the *P. falciparum* life cycle [35]. We performed RNA-Seq differential expression analysis between parasites with non-genetic halofuginone drug resistance and their parental Dd2 strain.

To disentangle stage-specific from phenotypic differential expression, we compared our RNA-Seq expression data with an RNA-Seq time course with 4 h resolution (KM Broadbent *et al.*, submitted). We determined which stage in the IDC time course each of our RNA-Seq libraries most closely resembled (Figure S9b in Additional file 15); the untreated Dd2 library most strongly correlated with the 34 hpi time point while the Dd2-Induced 1 and Dd2-Induced 2 libraries most strongly correlated with the 38 hpi time point. Next, we performed *in silico* differential expression analysis to generate a list of genes differentially expressed based on the variation in stage inherent in *P. falciparum* experimentation. By creating a linear fit between the stage controls, from the time course, and our halofuginone-induced parasites (Figure S9c in Additional file 15), we eliminated stage-specific artifacts and were left with a list of seven differentially expressed genes (Figure S9d in Additional file 15). These differentially expressed genes represent a mixture of genes differentially expressed between Dd2 and the 3D7 parasite used for the control time course and genes potentially involved in halofuginone-induced resistance.

## Discussion and conclusions

We previously identified the cPRS as the target of febrifugine derivatives such as halofuginone and halofuginol in *P. falciparum* (JD Herman *et al.*, submitted). In the present study, we took a genomic evolution approach using metagenomic sequencing to investigate the temporal emergence of drug resistance and discovered a novel non-genetic adaptation that occurs rapidly upon exposure to halofuginone. This precedes either SNP emergence in *cPRS* or amplification of the *cPRS* locus, both of which occur after prolonged, stepwise selection. By profiling polar metabolites, we found that re-calibrating proline homeostasis is central to halofuginone short-term induced resistance and a novel interplay of non-genetic and genetic mechanisms contributing to malaria drug resistance.

Whole genome sequencing of HFGRII at different time points has provided deep insight into the relative fitness of *cPRS* mutations and amplification. By looking at snap shots of our *in vitro* evolution experiments, we saw the rise and fall of *cPRS* mutant and WT alleles. The clonal competition between WT amplified *cPRS* and C1444T *cPRS* single-copy clones observed between the 41st and 58th generation argues that *cPRS* amplifications are more fit at higher halofuginone drug pressures than the HFGRII *cPRS* mutants. As a pulsed-drug *in vitro* selection, the advantage of amplified *cPRS* alleles represents clonal competition in the presence and absence of halofuginone. In the light of our findings in JD Herman *et al.,* (submitted) that cPRS mutant proteins have decreased affinity for their substrate proline, our genomic approach further supports our suspicion that *cPRS* mutations confer a competitive fitness cost and validates targeting *cPRS* as a viable and promising strategy for the development of future antimalarials.

Using our induced resistance parasite lines, we sought to understand the underlying non-genetic mechanism of resistance to halofuginone. We found that proline was increased 20- to 30-fold in the cytoplasm of *P. falciparum* parasites with short-term induced resistance (Figure 3; Figure S5a in Additional file 8). The pattern of proline and proline-related amino acid transport (Figure 5C) is highly similar to that performed by the high-affinity mammalian neuronal L-proline transporter SLC6A7 (PROT) present in gabaergic synapses [36] and epithelial L-proline transporter SLC6A20 (IMINO) [37]; in fact characterization of hPROT found pipecolic acid was the strongest competitive inhibitor of L-proline transport among six analogues [36]. These data are highly suggestive of a proline-specific importation mechanism underlying halofuginone-induced resistance. We are currently investigating *P. falciparum* SLC6A homologues for their role in proline transport and halofuginone-induced resistance. We have determined that there are no genic mutations in the halofuginone long-term selected or short-term induced parasites in the SLC6A homologues. Alternative explanations include reduced catabolism of proline to arginine [38] or alterations in proline export, as has been reported in prokaryotes [39].

Thus, our metabolomics analysis is highly suggestive that non-genetic halofuginone resistance is caused by a proline-specific transport mechanism. Comparison of our steady-state measurements of total iRBCs and purified parasites implies that this transporter is located on the parasite or parasitophorous vacuolar membrane. However, further experimental work will be required to confirm this hypothesis.

The implication that proline transport is involved in halofuginone-induced resistance led us to investigate the involvement of the *clag* multi-gene family. We see no cell cycle-independent changes in *clag* gene expression in qPCR or RNA-Seq studies (Additional file 15). Our stage-controlled RNA-Seq analysis revealed seven differentially expressed genes. However, we cannot determine whether they represent genes involved in non-genetic halofuginone resistance or genes differentially expressed between the *P. falciparum* Dd2 and 3D7 strains. None have clear involvement in proline biogenesis, transport, or protein degradation. Additionally, none are homologous to the SLC6A family of amino acid transporters. Thus, we have not found strong evidence that differential RNA expression can explain non-genetic halofuginone resistance.

We believe the stable upregulation of intracellular amino acid homeostasis represents a new and potentially general mechanism of drug resistance in malaria. The non-genetic alteration of proline amino acid transport is a strain-independent stable mechanism of halofuginone resistance. We as a field have focused on identifying genetic mechanisms of malarial drug resistance with the assumption that they are the primary contributors to stable and heritable resistance. Our evidence of an *in vitro* stable non-genetic mechanism of resistance questions this assumption. Though the alteration of intracellular proline we found will likely not be a pan-drug resistance mechanism, it suggests that metabolic mechanisms of drug resistance/tolerance exist in *P. falciparum*. The drug resistance literature in the field of cancer biology has also begun to appreciate metabolic methods of drug resistance. Changes in central carbon metabolism [39], including the Warburg effect [40], and in amino acids involved in oxidative stress responses [41,42] have been demonstrated to confer drug resistance in tumor cells. Combining proteomic and metabolomic approaches will be essential to understanding the metabolomic basis of malaria drug resistance.

The non-genetic proline-based resistance we have found in halofuginone-induced parasites differs from previous instigations of amino acid regulation in *P. falciparum.* Much previous work in *P. falciparum* amino acid homeostasis has focused on the partially conserved amino acid starvation pathway [23,43,44], which results in translational inhibition and a hibernation-like state [44]. We believe that this stable non-genetic resistance is not an immediate starvation response, but a re-regulation of metabolism. Our group has also shown that halofuginone activates the amino acid starvation pathway within 90 minutes (JD Herman *et al.*, submitted). Unlike the amino acid starvation pathway, in which signal transduction occurs within minutes to hours, halofuginone induction of increased intracellular proline occurs after multiple generations of growth. In addition to the temporality, the two biological phenomena differ in their effect on intracellular amino acid levels. Babbit *et al*. [44] saw no difference in either proline or isoleucine levels between isoleucine-fed and isoleucine-starved parasites. Based on temporality and amino acid homeostatic concentrations, we thus posit that halofuginone-induced resistance represents a separate biological phenomenon resulting in long-term upregulation of proline homeostasis.

**Figure 6:**
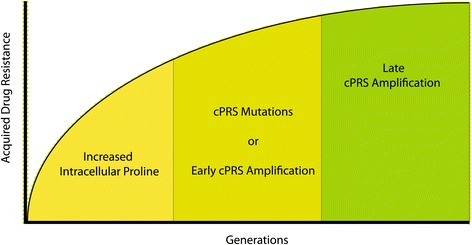
**A model of step-wise acquisition of halofuginone drug resistance.**

To explain the multistep mechanisms of halofuginone drug resistance, we propose a multi-step adaptation process that maximizes fitness in increasing concentrations of halofuginone. In the first phase of our *in vitro* evolution, resistance is acquired by altering cellular amino acid homeostasis; specifically, intracellular proline levels are elevated in response to halofuginone pressure. This phenomenon is analogous to the induced resistance phenotype we found with low-dose constant drug treatment. The second phase of drug resistance represents alterations in *cPRS* in mutually exclusive ways. We have witnessed either target-site mutations or amplification in this second temporal phase. However, the third phase of drug resistance encompasses amplification of the WT target loci in the presence of increasing selective drug pressure regardless of prior mutation (Figure 6). This result implies that a mutation in *cPRS* renders the parasite less fit. Our attempts at allelic replacement of the WT genomic copy with a single copy of the C1444T *cPRS* allele have been unsuccessful; all clones that have been isolated after transfection contain multiple copies of the *cPRS* gene with both WT and mutant alleles present and all have elevated intracellular proline levels (data not shown).

Our understanding of the sequential acquisition of halofuginone resistance is contrary to the commonly used model of compensatory mutations. In such a model, the evolution of drug resistance results in organisms with decreased fitness in the absence of drug pressure [45,46]. Additional ‘compensatory’ mutations are acquired to rescue the fitness of the drug-resistant organism. Rather than finding post-*cPRS* compensatory mutations, we found that metabolic cellular adaptations preexisted *cPRS* mutations under halofuginone selective pressure. This model has larger implications for understanding the initial steps of evolutionary adaptation.

Mapping resistance mechanisms is also essential to furthering malarial drug discovery. Identifying resistance mechanisms will inform the future usefulness of an antimalarial compound. Further, this model of halofuginone resistance will inform the rational design of combination therapy, most obvious of which is the combination of febrifugine derivatives with inhibitors to the yet-to-be identified proline transport mechanism. With this combination, we could block the early non-genetic adaption that precedes the evolution of genetic resistance. Thus, rather than targeting the resistant enzyme and making it less fit [47], one could propose a strategy to eliminate the evolutionary path that would lead to target site mutations and amplification at the *cPRS* locus.

Our whole-genome population sequencing approach revealed a non-genetic stable resistance that we have tracked to amino acid homeostasis. We believe genomic technologies allow exploration beyond the obvious next step hypothesis and unlock a powerful new level of insight and progress for the field of malariology and infectious disease research in general.

## Materials and methods

### Metagenomic whole-genome sequencing of halofuginone populations selected *in vitro*

The goal of this experiment was to define the evolutionary trajectory of halofuginone drug resistance in a *P. falciparum in vitro* long-term culture experiment. We performed two replicate long-term intermittent pressure selections in order to increase the concentration a clonal strain of Dd2 parasites could tolerate; we named the two strains HFGRII and HFGRIII. We started with 10× EC50 (7 nM) and subsequently increased the pressure to 30× (21 nM), 60× (42 nM), and then 200× (140 nM) (Additional files 1, 2, and 3). The notation HFGRII 2-10x signifies the selection HFGRII was selected two times with 10× EC50 (7 nM). At each step along the selection, we isolated genomic DNA from the entire evolving population. We chose six time points for HFGRII and seven time points for HFGRIII spaced across the approximately 60 generations of halofuginone selection in order to track the multiple genetic mechanisms that may contribute to halofuginone resistance.

Genomic DNA extracted from bulk population cultures of the two independent lines selected for halofuginone resistance was extracted and sheared with a Covaris S220 Focused-ultrasonicator (Covaris, Woburn, MA, USA). Illumina-compatible libraries were prepared on the Apollo 324 (WaferGen Biosystems, Fremont, CA, USA) and sequenced on an Illumina HiSeq 2000 (Illumina, San Diego, CA, USA). *P. falciparum* populations were sequenced with the goal of reaching over 60× average fold-coverage across the genome.

### Metagenomic time series analysis to identify segregating sites

We used a two-stage process to distinguish real mutations from sequencing and alignment errors in the metapopulation time-series sequence data. First, we used the GATK (Broad Institute, Cambridge, MA, USA) to obtain a permissive list of candidate loci and the number of supporting reads for each allele at each time-point. We used the haploid mode of GATK’s Unified Genotyper with a minimum confidence threshold phred score of 4 to obtain a permissive set of SNPs and small indels. We further filtered this set of candidates based on two fundamental assumptions: first, the two independent replicate populations should not share exactly the same mutations; and second, the frequencies of a real mutation should be correlated through time (positive autocorrelation), while sequencing errors should be uncorrelated at different time-points (negative or zero autocorrelation).

Thus, we 1) discarded all sites with an average coverage depth less than 10× across the time-points or no coverage at more than three time-points, 2) discarded all sites with an average frequency above 1% in the non-focal population, 3) discarded all sites with a negative or zero autocorrelation, and 4) discarded all mutations supported by fewer than 10 total reads or with a maximum frequency of less than 10%. Further, we noticed that the frequencies of alternative alleles in HFGRII at time-point 2-30× (27th generation) were aberrant despite deep coverage and we discarded this time-point from the analysis due to potential contamination.

### Metagenomic time series analysis to identify selective sweeps

We took the unfiltered list of candidate loci called by the Unified Genotyper (GATK) and looked for mutations that began at a frequency near zero at the first time-point and ended at a frequency near one at the last time-point.

### Metagenomic time series analysis to identify hitchhiking mutations along with *cPRS* mutation in HFGRII

We searched for mutations that hitchhiked with the known *cPRS* mutation C1444T in HFGRII. To find hitchhiking mutations, we sorted the unfiltered list of candidate loci called by the Unified Genotyper (GATK) by the Euclidean distance between their allele frequency trajectories and the frequency trajectory of the focal mutation.

### Quantitative PCR - relative copy number analysis

Genomic DNA was prepared from saponin-lysed *P. falciparum* parasites using the Qiagen Blood Mini- or Midi Kits (Qiagen, Venlo, Limburg, the Netherlands). qPCR analysis was performed on an Agilent 7900HT Fast Real-Time System (Agilent Technologies, Santa Clara, CA, USA) using unlabeled primers and Power SYBR Green master mix. Primers used for copy number analysis are listed in Additional file 16 (control locus primers) and Additional file 17 (target locus primers). Copy number was calculated using the ΔΔCt method included in SDS version 2.3.2 as described in the Applied Biosystems User Bulletin 2.

### *In vitro* drug sensitivity and dose-response analysis by SYBR Green staining

The SYBR Green I method was used as previously described [48]. In brief, we grew *P. falciparum* parasites for 72 hours in 384-well plates at 1% hematocrit and 1% starting parasitemia. Growth was assessed by SYBR Green staining of parasite DNA. All dose-response assays were carried out with 12-point curves in technical triplicate. DMSO stocks of drugs used were dispensed by HP D300 Digital Dispenser (Hewlet Packard, Palo Alto, CA, USA). Fluorescence measurements were collected on a SpectraMax M5 (Molecular Devices, Sunnyvale, CA, USA) and analyzed on GraphPad Prism version 5 (GraphPad Software, La Jolla, CA, USA) and EC50 values were determined with the curve-fitting algorithm Log(inhibitor) versus Response - Variable slope.

### *In vitro* drug sensitivity and dose-response analysis by tritiated hypoxanthine assay

Halofuginone dose-response assays were performed as described by Desjardins *et al*. [49] using the initial drug resistance selected HFGRII parasites.

### Parasite culture

Malaria culture was performed as described by Trager and Jensen [50]. Dd2 is a chloroquine-resistant *in vitro* cultured parasite cloned from the Laos derived W2-MEF parasite obtained from MR4 (ATCC, Manassas, VA, USA).

### Flow cytometry

Flow cytometry of *P. falciparum*-infected erythrocytes was carried out based on SYBR Green I staining of parasite nuclei as previously described [51]. Modifications to the Bei *et al*. [51] protocol include staining of iRBCs with SYBR Green I at a 1:2,000 concentration, and acquisition of non-single cells pre-filtered for cells that fell on the y = x line in a plot of forward scatter area versus height. All flow cytometry was collected on a MACSQuant Flow Cytometer (Miltneyi Biotec Inc., San Diego, CA, USA) and analyzed with MacQuantify and FlowJo 8.8.6 software (Tree Star, Ashland, OR, USA).

### LC-MS analysis of amino acids and polar metabolites

Highly synchronous (within 4 hours) early schizonts were magnetically purified with MACS CS columns (Miltneyi Biotec Inc., San Diego, CA, USA). A small aliquot was also made for flow cytometry. The rest of the purified samples were divided into two equal volumes: one for saponin lysis (0.025%) and one for whole iRBC extraction. Each sample was washed twice in phosphate-buffered saline, and then suspended in 10 μl phosphate-buffered saline (Life Technologies, Carlsbad, CA, USA). Polar metabolites were extracted using nine volumes of 74.9:24.9:0.2 (v/v/v) acetonitrile/methanol/formic acid containing stable isotope-labeled internal standards (0.2 ng/μl valine-d8 (Sigma Aldrich, St. Louis, MO, USA); and 0.2 ng/μl phenylalanine-d8 (Cambridge Isotope Laboratories,Tewksbury, MA, USA)). Profiles of amino acids were measured using LC-MS as described previously [52]. Briefly, positive ionization, multiple reaction mode (MRM) data were acquired using a 4000 QTRAP triple quadrupole mass spectrometer (AB SCIEX, Framingham, MA, USA) coupled to an 1100 Series pump (Agilent) and an HTS PAL autosampler (Leap Technologies, Carrboro, NC, USA). Cell extracts (10 μl) were injected onto a 150 × 2.1 mm Atlantis HILIC column (Waters, Milford, MA, USA). The column was eluted isocratically at a flow rate of 250 μl/minute with 5% mobile phase A (10 mM ammonium formate and 0.1% formic acid in water) for 1 minute followed by a linear gradient to 40% mobile phase B (acetonitrile with 0.1% formic acid) over 10 minutes. The ion spray voltage was 4.5 kV and the source temperature was 450°C. MultiQuant 1.2 software (AB SCIEX) was used for automated peak integration and metabolite peaks were manually reviewed for quality of integration and compared against a known standard to confirm identity. Stable isotope-labeled internal standards were used to eliminate samples with poor data quality. Metabolite peaks signals were total-signal normalized with all 115 metabolites. Pearson correlation analysis was performed in prism and heat maps were generated with Gene-e (Broad Institute).

### Quantitative PCR - gene expression analysis

Total RNA was extracted using Trizol (Life Technologies, Carlsbad, CA, USA) according to the manufacturer’s instructions, DNAse-treated, and re-purified with Qiagen RNeasy mini columns. First strand cDNA synthesis was performed using SuperScript III (Life Technologies) following the manufacturer’s instructions. The absence of contaminating DNA and the success of the reverse transcriptase reaction were confirmed by comparing qPCR of Rt + and Rt- with the control seryl tRNA synthetase primer set in quadruplicate; samples were run on and ABI 7900 HT and fold expression calculated using the ABI software suite SDS 2.3.2. cDNA concentrations were normalized to SerRS Ct values to minimize biases in PCR efficiency. Samples were run in quadruplicate with two control primer sets for validation of expression analysis. PCR amplification was performed as follows: 15 minutes at 95°C followed by 40 cycles of two-step amplification of 94°C for 30 s and 52°C for 30 s. All primers used for expression analysis (Additional files 16, 17, and 18) were validated for specificity and efficiency under the same PCR conditions.

### RNA-Seq expression analysis

Synchronized late schizont *P. falciparum* Dd2 untreated and Dd2 Induced 1 and Dd2 Induced 2 parasites were lysed with saponin. Total parasite RNA was purified with Qiagen RNeasy mini columns. The polyA tagged RNA was purified with PrepXTM PolyA Protocol on an Apollo 324 Library Prep System (Wafergen). Strand-specific RNA-Seq libraries were assembled with the PrepX mRNA Library Protocol and quantified with the Kapa NGS Library Quantification Kit. Libraries were sequenced on an Illumina HiSeq 2000 using 101-bp, paired-end read technology.

Raw reads were aligned using TopHat 2.0.1 against the *P. falciparum* 3D7 PlasmoDB version 10.0 genome. Because so many genes in the falciparum genome are homologous, very stringent alignment parameters were used: -r 300 -mate-std-dev 100 -library-type fr-firststrand -i 70 -I 5000 -read-mismatches 0 -segment-mismatches 0 -max-segment-intron 5000 -max-coverage-intron 5000 -b2-very-sensitive -read-gap-length 0 -read-edit-dist 0 -read-realign-edit-dist 0 -max-deletion-length 0 -max-insertion-length 0 -max-multihits 2 -no-mixed -no-discordant to be consistent with the 3D7 RNA-Seq 4-h time course from Broadbent *et al.* (submitted). Roughly one-third of reads could be aligned using these settings.

To determine the exact stage of the Dd2 control and experimental expression profiles, correlation analyses were performed using Python, numpy, scipy.stats.stats, and plotted using matplotlib. Spearman correlation was calculated between each of the three Dd2 RNA-Seq libraries and the nine 3D7 RNA-Seq time-points separated throughout the 48-h life cycle (KM Broadbent *et al.*, submitted).

Gene expression was quantified using Cufflinks 2.2, with annotations from PlasmoDB version 10. Differential expression of genes in the Dd2 Induced 1 and Dd2 Induced 2 samples were calculated with respect to the Dd2 untreated sample. To control for stage-specific differentially expressed genes, we calculated differential expression between the 3D7 time-points that most strongly correlated with the Dd2 induced and Dd2 untreated samples. Distributions of log transformed fold changes of the Dd2 and corresponding 3D7 time-points were analyzed using R, and plotted using ggplot2. Outliers from the linear fitting that were shared between the two biological replicate inductions with more than 10 reads per gene model in each RNA-Seq library were determined to be differentially expressed.

### Data availability

BAM files for all genomic and transcriptomic analysis are accessible on NCBI’s Sequence Read Archive with BioProject accession ID PRJNA167166.

## Additional files

Additional file 1: Table S1. Sequenced time-points from the HFGRII *in vitro* evolution experiment. Additional file 2: Table S2. Sequenced time-points from the HFGRIII *in vitro* evolution experiment. Additional file 3: Figure S1. Diagram of long-term drug resistant selection experiments, demonstrating the stepwise intermittent selection protocol used for long-term evolutionary studies of the halofuginone resistance trajectory in HFGRII and HFGRIII. Additional file 4: Table S3. Filtering scheme for metagenomic sequence analysis of HFGRII and HFGRIII. Additional file 5: Table S4. Filtered variants from metagenomic sequence analysis of HFGRII and HFGRIII. Additional file 6: Figure S2. Copy number variation frequency spectrum at the *cPRS* locus of the 55th generation of HFGRIII (2-200× ) cloned parasites. Of the parasites segregating at the 55th generation of HFGRIII, 71% have three WT copies of *cPRS*. We tested 14 parasites cloned by dilution from the HFGIII 55th generation for *cPRS* amplification. Copy number variation was determined by ∆∆Ct qPCR using the 3′ *cPRS* assay. Seryl-tRNA synthetase served as the control gene and all copy number variation quantification was made in reference to a single *cPRS* copy Dd2 parasite. Additional file 7: Table S5. Correlation between *cPRS* copy number and drug resistance. R^2^ Pearson correlation coefficients of the relationship between *cPRS* copy number and the EC50 values of 11 antimalarial compounds. The parasites used for this analysis were HFGRIII 2-200× clone H3 (one copy), H6 (two copies), and A8 (three copies). R^2^ values range from 0 to 1, representing little to strong correlation, respectively. *Two-tailed *t*-test *P*-value <0.05. Additional file 8: Figure S5. Induction of halofuginone resistance in HB3 confirms proline-dependent induction of halofuginone resistance is not strain specific. **(a)** After eight generations of exposure to 4× EC50 of halofuginone (2.8 nM), HB3-induced parasites recrudesced and returned 20- to 30-fold resistant to halofuginone treatment. (b,c) Of the 19 proteogenic amino acids assayed, only proline concentrations were increased in HB3-induced parasites in saponin-released parasites (b) and purified iRBCs (c). EC50s were determined by SYBR Green growth assay. Fold increase in amino acid concentration upon induction or selection is expressed as a ratio of induced/selected line measurement over parental Dd2 line measurement. Amino acid levels of *P. falciparum* parasites were quantified from the normalized integrated peak intensity from LC-MS analysis. Error bars denote standard deviation. Additional file 9: Figure S3. Induced Dd2 parasites show no evidence of genetic modifications at the *cPRS* locus. **(a)** Sanger sequencing across the *cPRS* allele revealed that induced and untreated Dd2 strains have WT *cPRS* alleles. The codon where the HFGRI (T14445A) and HFGRII (C1444T) mutations occurred is highlighted in yellow. **(b)** Expression of *cPRS* in synchronous early schizonts is invariant between untreated and induced Dd2 parasite lines. Additional file 10: Table S6. SNPs called between whole-genome sequencing of Dd2 untreated and Dd2 Induced 1 and Dd2 Induced 2 lines. Additional file 11: Figure S4. Halofuginone short-term induced long-term selection lines specifically upregulate intracellular proline concentration as measured in magnetically purified iRBCs. **(a,b)** Of the 19 proteogenic amino acids assayed, only proline concentrations were increased in Dd2 induced (a) and Dd2 long term selected (b) HFGRII and HFGRIII parasites within iRBCs. iRBC proline concentrations increased 4- to 6-fold compared with 20- to 30-fold in the corresponding saponin-released parasite samples. Fold-increase in amino acid concentration upon induction or selection is expressed as a ratio of induced/selected line measurement over parental Dd2 line measurement. Amino acid levels of RBCs infected with *P. falciparum* parasites were quantified from the normalized integrated peak intensity from LC-MS analysis. Error bars denote standard deviation. Additional file 12: Figure S6. Early dynamics of proline and halofuginone resistance in Dd2-induced parasites. After eight generations of exposure to 4× EC50 of halofuginone (2.8 nM), Dd2-induced parasites recrudesced. **(a)** As early as five generations post-recrudescence, Dd2-induced parasites had elevated intracellular proline levels that remained constantly elevated for an additional 55 generations. Proline levels of saponin-released *P. falciparum* parasites were quantified from the normalized integrated peak intensity from LC-MS analysis. **(b)** Dd2 parasites five generations post-recrudescence had also reached their maximum halofuginone resistance (EC50s 25 to 30 nM). Fifty-five generations later, the halofuginone dose-response in Dd2-induced parasites was unchanged. Error bars in (b) denote 95% confidence interval. Additional file 13: Figure S7. Dose-dependent inhibition of *P. falciparum* growth is dependent on the concentration of free proline. SYBR Green dose response assays were performed with untreated (WT) Dd2 parasites that were put in media with four different proline concentrations at the start of the 72-h assay. The proline concentration of different *in vitro* culture medias is expressed relative to unmodified RPMI media. Error bars reflect standard deviations of triplicate experiments. Additional file 14: Figure S8. Correlation of full metabolite profile with proline concentrations in halofuginone short-term induced and long-term selected parasites. Of the 115 polar metabolites measured, only pipercolic acid and *cis*/*trans*-hydroxyproline concentrations are highly correlated with cytosolic proline concentrations in short-term induced Dd2 parasites, short-term induced HB3 parasites, and long-term selected Dd2 lines HFGRI, II, and III. This heat map represents the correlation coefficients (r-values) between cytosolic concentrations of metabolites with that of proline in three collections of wild-type and modified parasites: short-term induced Dd2 parasites, short-term induced HB3 parasites, and long-term selected Dd2 HFGR lines. High correlation (r = 1) is represented by red while low correlation (r = -1) is represented by blue. Additional file 15: Figure S9. Expression analysis of Dd2-induced parasites. (a) qPCR-based expression analysis of the *clag* family of transport-involved genes demonstrates that their differential expression is not involved in Dd2 induced halofuginone resistance. Relative expression was calculated with the ddCt method using SerRS as control and in reference to a stage-matched untreated Dd2 parasite. FBP-Aldolase and ProRS (5′) are provided as additional controls. Results represent eight biological replicate measurements (each data point) each determined by technical quadruplicate. (b) Spearman correlation was used to directly identify the stage of the three Dd2 RNA-Seq libraries assembled. (c) Log-transformed fold change of replicate Dd2 inductions (Dd2-Induced 1 and Dd2-Induced 2) (y-axis) was graphed with the log-transformed fold change from a 3D7 RNA-Seq control time course (x-axis) between corresponding stages (40 vs 36 hours post-infection ). By identifying outliers from this linear fitting, (d) we determined genes that are differential expressed between Dd2 induced and untreated parasites independent of stage-specific expression. Additional file 16: Table S7. Quantitative PCR control primers for ∆∆Ct analysis. Additional file 17: Table S8. Quantitative PCR primers for the *cPRS* target locus analysis. Additional file 18: Table S9. Quantitative PCR primers for the *clag* gene family expression analysis.

## Abbreviations

cPRS: cytoplasmic prolyl-tRNA synthetase
GATK: Genome Analysis Toolkit
HFGRII/III: long-term halofuginone drug resistance selection II/III
hpi: hours post-infection
iRBC: infected red blood cell
LC-MS: liquid crystallography-mass spectrometry
PCR: polymerase chain reaction
qPCR: quantitative PCR
SNP: single-nucleotide polymorphism
WT: wild type
