# Supplementary material for: A genomic and evolutionary approach reveals non-genetic drug resistance in malaria
Source: Genome Biol. 2014 Nov 14;15(11):511. doi: 10.1186/s13059-014-0511-2 (PMC4272547; doi:10.1186/s13059-014-0511-2)
Supplement: Additional file 1: Table S1. — Sequenced time-points from the HFGRII in vitro evolution experiment. [file 13059_2014_511_MOESM1_ESM.doc]

| **HFGRII Population Time Points Sequence** | **Time (Days)** | **Generations** | **Drug Pressure (EC50)** | **Drug Pressure (nM)** | **Fold Coverage** | **SRA /Biosample Number** |
| --- | --- | --- | --- | --- | --- | --- |
| 2-10x | 36 | 18 | 10x | 7 | 185 | SAMN03135254 |
| 1-30x | 46 | 23 | 30x | 21 | 263 | SRX158287 |
| 2-30x | 54 | 27 | 30x | 21 | 141 | SRX158288 |
| 2-60x | 68 | 34 | 60x | 42 | 232 | SRX158286 |
| 1-200x | 82 | 41 | 200x | 140 | 235 | SRX158283 |
| 2-200x | 100 | 50 | 200x | 140 | 181 | SAMN03135255 |
