# Supplementary material for: A genomic and evolutionary approach reveals non-genetic drug resistance in malaria
Source: Genome Biol. 2014 Nov 14;15(11):511. doi: 10.1186/s13059-014-0511-2 (PMC4272547; doi:10.1186/s13059-014-0511-2)
Supplement: Additional file 2: Table S2. — Sequenced time-points from the HFGRIII in vitro evolution experiment. [file 13059_2014_511_MOESM2_ESM.doc]

| **HFGRIII Population Time Points Sequence** | **Time (Days)** | **Generations** | **Drug Pressure (EC50)** | **Drug Pressure (nM)** | **Fold Coverage** | **SRA /Biosample Number** |
| --- | --- | --- | --- | --- | --- | --- |
| 2-10x | 36 | 18 | 10x | 7 | 12 | SAMN03135257 |
| 1-30x | 46 | 23 | 30x | 21 | 263 | SRX158289 |
| 1-60x | 64 | 32 | 60x | 42 | 241 | SRX158284 |
| 2-60x | 72 | 36 | 60x | 42 | 246 | SRX158285 |
| 1-200x | 90 | 45 | 200x | 140 | 240 | SRX200273 |
| 2-200x | 110 | 55 | 200x | 140 | 92 | SAMN03135260 |
| 3-200x | 117 | 58.5 | 200x | 140 | 92 | SRX158282 |
