# Supplementary figures and images for: A genomic and evolutionary approach reveals non-genetic drug resistance in malaria
Source: Genome Biol. 2014 Nov 14;15(11):511. doi: 10.1186/s13059-014-0511-2 (PMC4272547; doi:10.1186/s13059-014-0511-2)

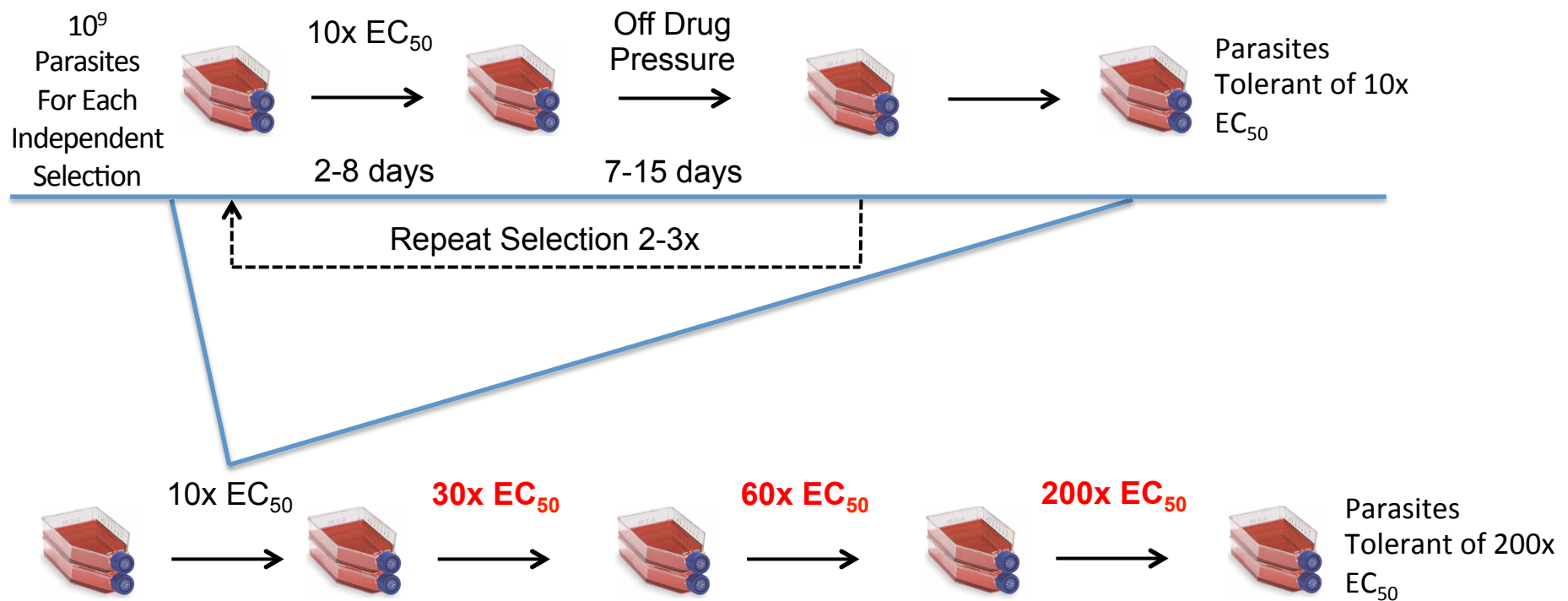

**Stepwise + Intermittent Selection**

Supplement: Additional file 3: Figure S1. — Diagram of long-term drug resistant selection experiments, demonstrating the stepwise intermittent selection protocol used for long-term evolutionary studies of the halofuginone resistance trajectory in HFGRII and HFGRIII. [file 13059_2014_511_MOESM3_ESM.pdf]

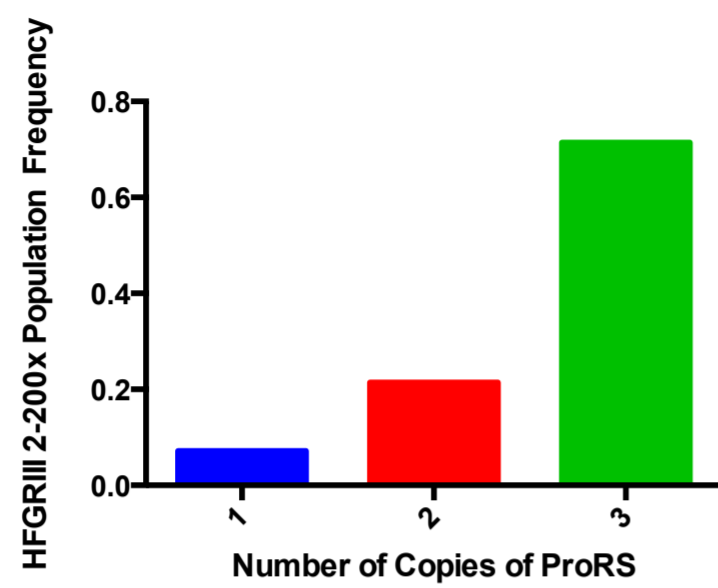

Supplement: Additional file 6: Figure S2. — Copy number variation frequency spectrum at the cPRS locus of the 55th generation of HFGRIII (2-200× ) cloned parasites. Of the parasites segregating at the 55th generation of HFGRIII, 71% have three WT copies of cPRS. We tested 14 parasites cloned by dilution from the HFGIII 55th generation for cPRS amplification. Copy number variation was determined by ∆∆Ct qPCR using the 3′ cPRS assay. Seryl-tRNA synthetase served as the control gene and all copy number variation quantification was made in reference to a single cPRS copy Dd2 parasite. [file 13059_2014_511_MOESM6_ESM.pdf]

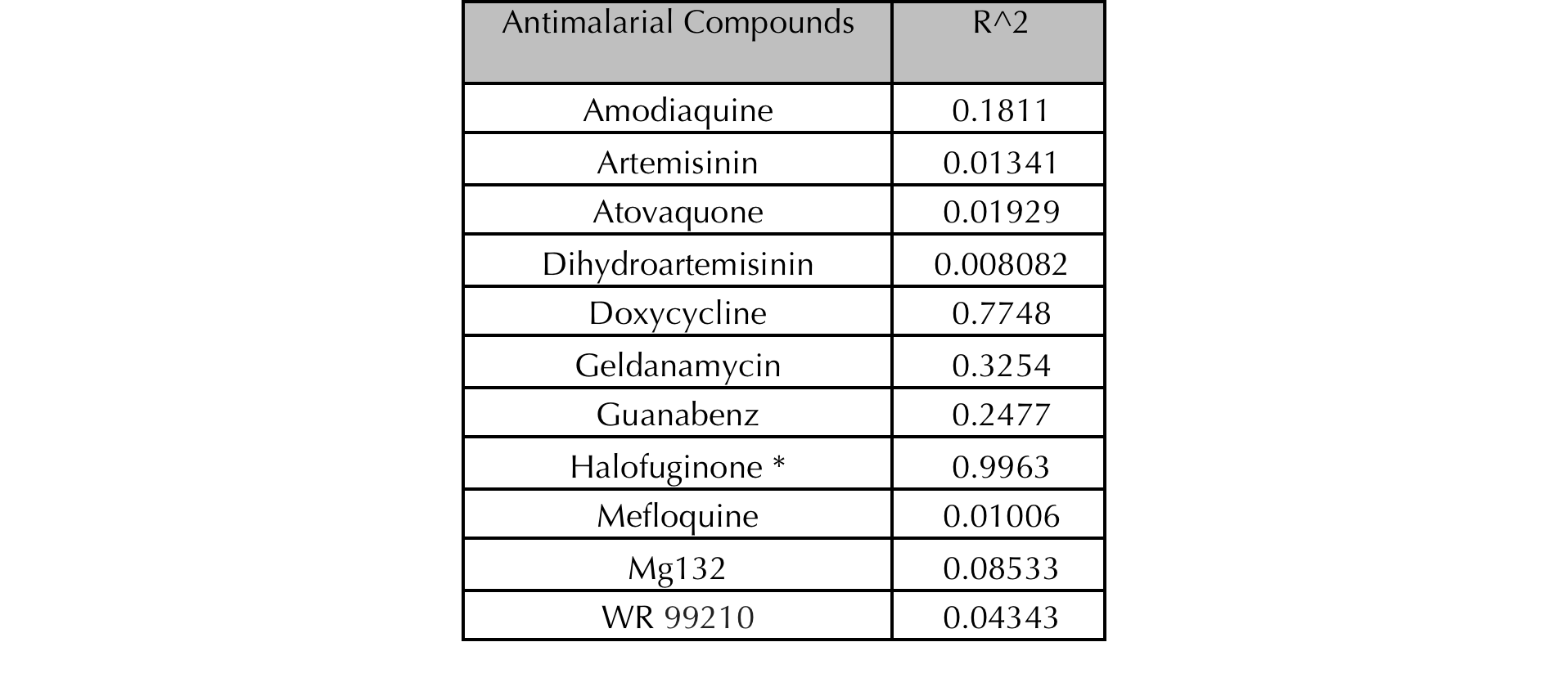

Supplement: Additional file 7: Table S5. — Correlation between cPRS copy number and drug resistance. R2 Pearson correlation coefficients of the relationship between cPRS copy number and the EC50 values of 11 antimalarial compounds. The parasites used for this analysis were HFGRIII 2-200× clone H3 (one copy), H6 (two copies), and A8 (three copies). R2 values range from 0 to 1, representing little to strong correlation, respectively. *Two-tailed t-test P-value <0.05. [file 13059_2014_511_MOESM7_ESM.doc]

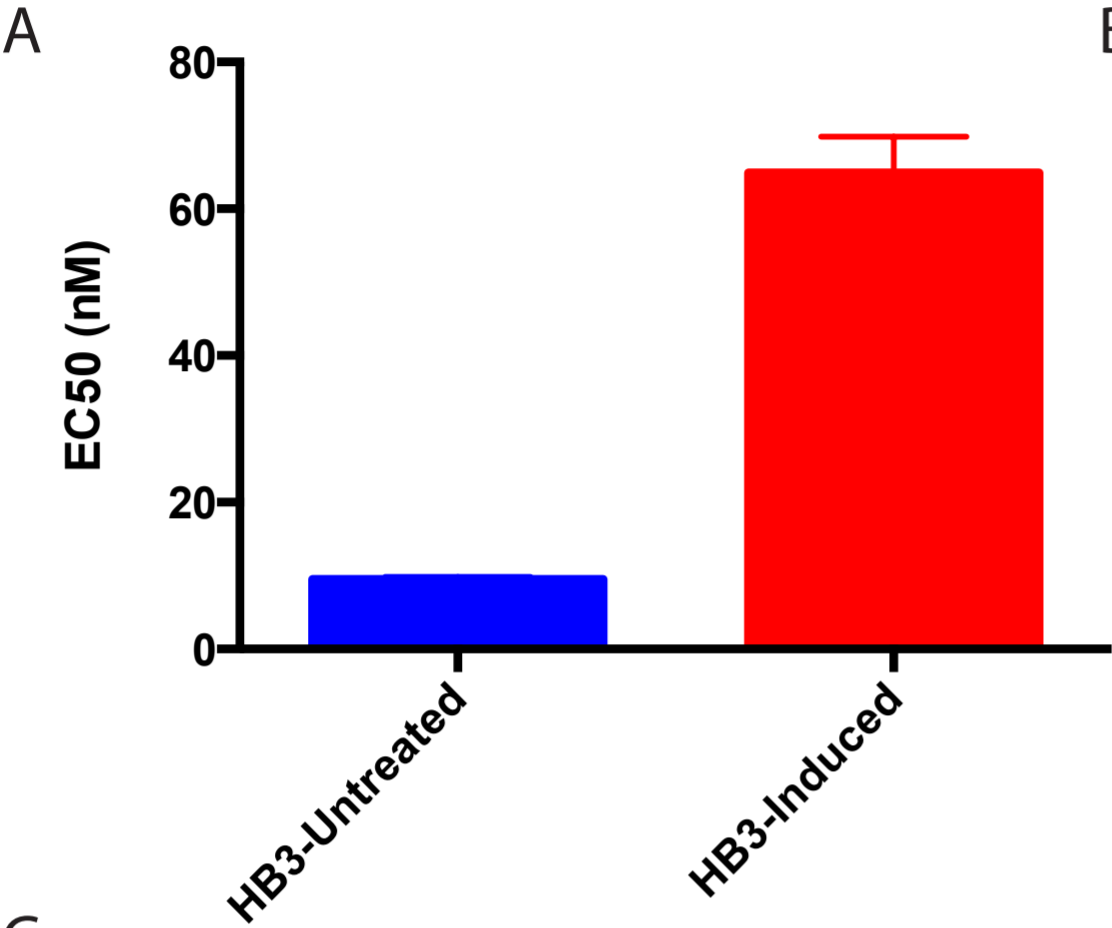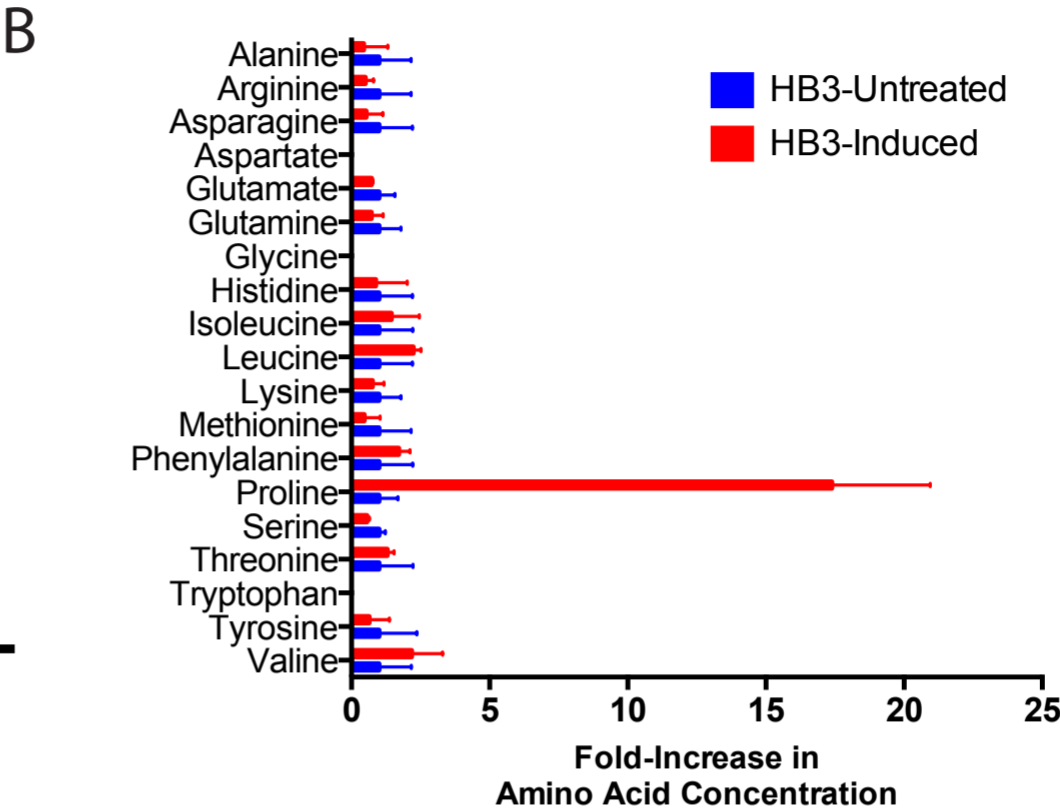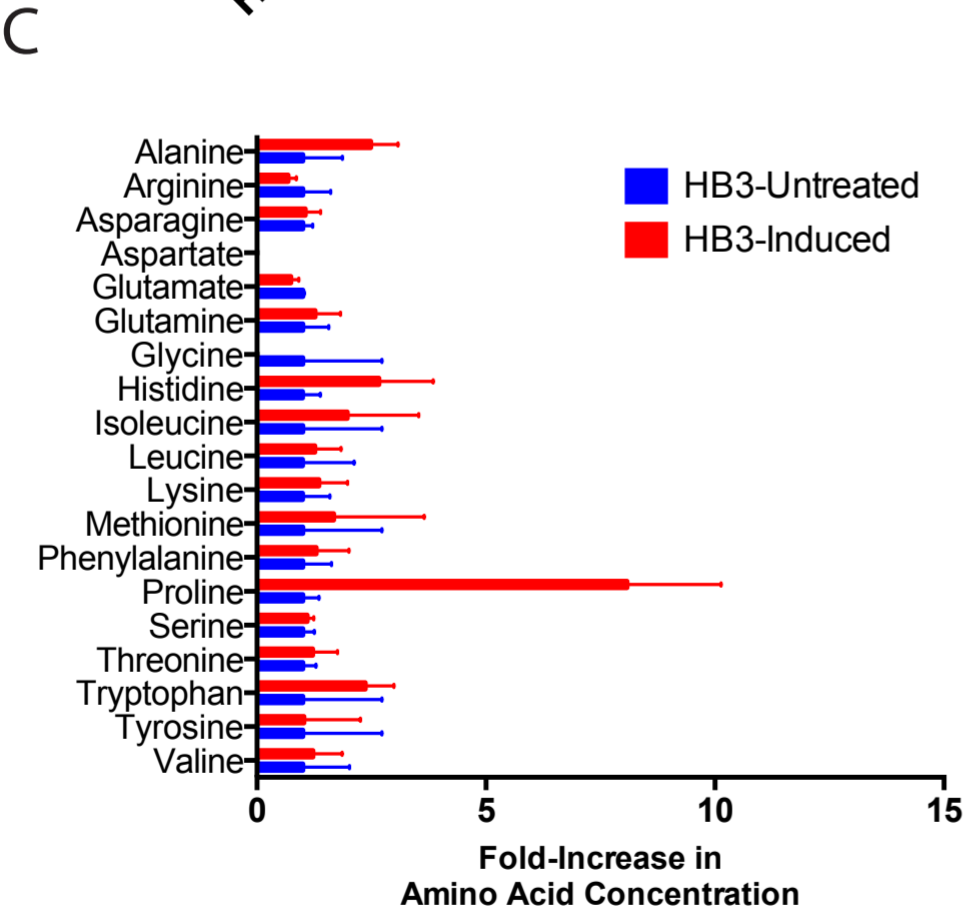

Supplement: Additional file 8: Figure S5. — Induction of halofuginone resistance in HB3 confirms proline-dependent induction of halofuginone resistance is not strain specific. (a) After eight generations of exposure to 4× EC50 of halofuginone (2.8 nM), HB3-induced parasites recrudesced and returned 20- to 30-fold resistant to halofuginone treatment. (b,c) Of the 19 proteogenic amino acids assayed, only proline concentrations were increased in HB3-induced parasites in saponin-released parasites (b) and purified iRBCs (c). EC50s were determined by SYBR Green growth assay. Fold increase in amino acid concentration upon induction or selection is expressed as a ratio of induced/selected line measurement over parental Dd2 line measurement. Amino acid levels of P. falciparum parasites were quantified from the normalized integrated peak intensity from LC-MS analysis. Error bars denote standard deviation. [file 13059_2014_511_MOESM8_ESM.pdf]

A

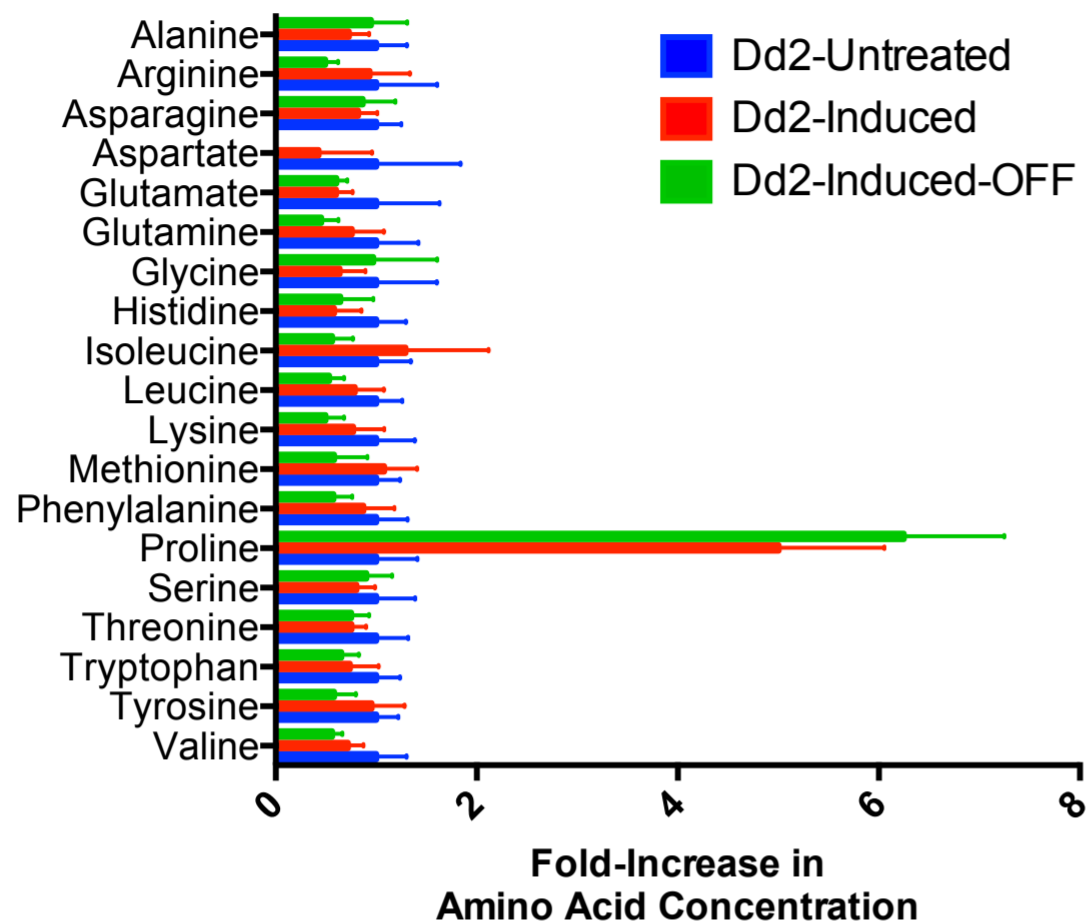

B

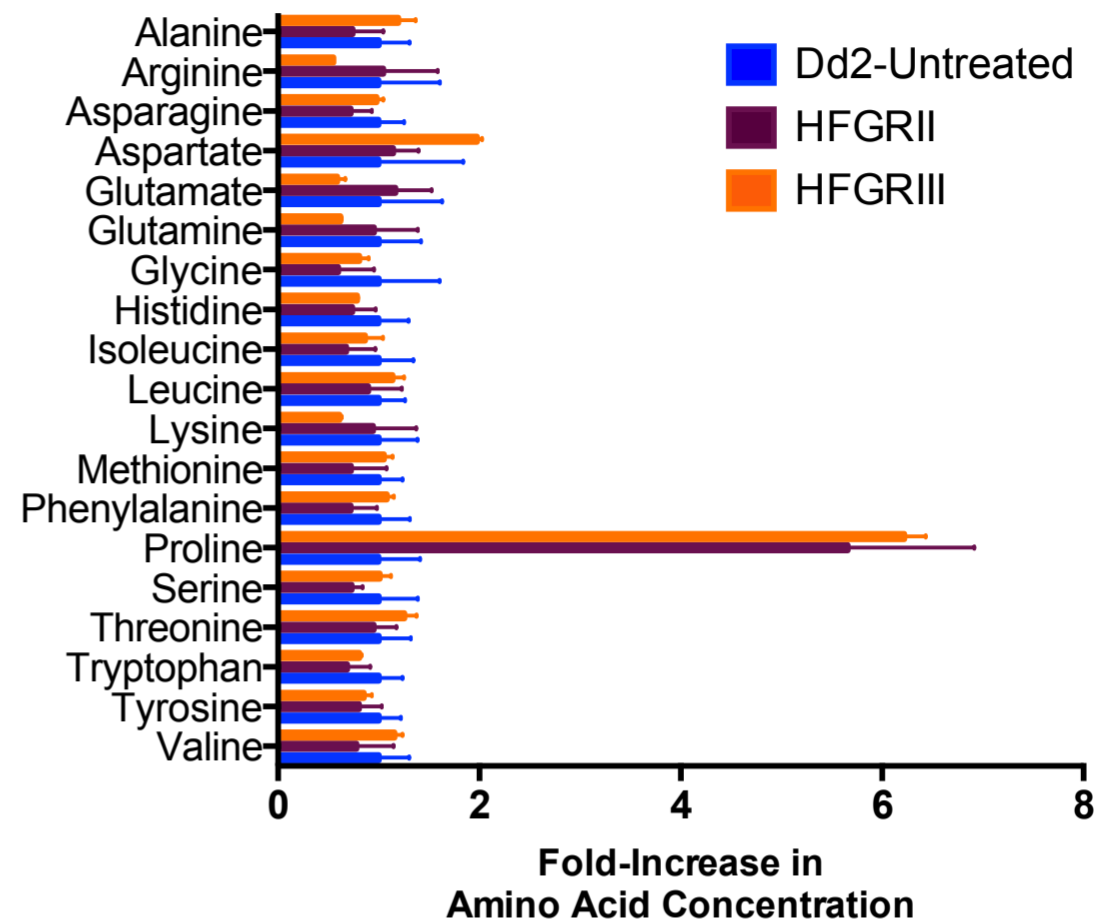

Supplement: Additional file 11: Figure S4. — Halofuginone short-term induced long-term selection lines specifically upregulate intracellular proline concentration as measured in magnetically purified iRBCs. (a,b) Of the 19 proteogenic amino acids assayed, only proline concentrations were increased in Dd2 induced (a) and Dd2 long term selected (b) HFGRII and HFGRIII parasites within iRBCs. iRBC proline concentrations increased 4- to 6-fold compared with 20- to 30-fold in the corresponding saponin-released parasite samples. Fold-increase in amino acid concentration upon induction or selection is expressed as a ratio of induced/selected line measurement over parental Dd2 line measurement. Amino acid levels of RBCs infected with P. falciparum parasites were quantified from the normalized integrated peak intensity from LC-MS analysis. Error bars denote standard deviation. [file 13059_2014_511_MOESM11_ESM.pdf]

A

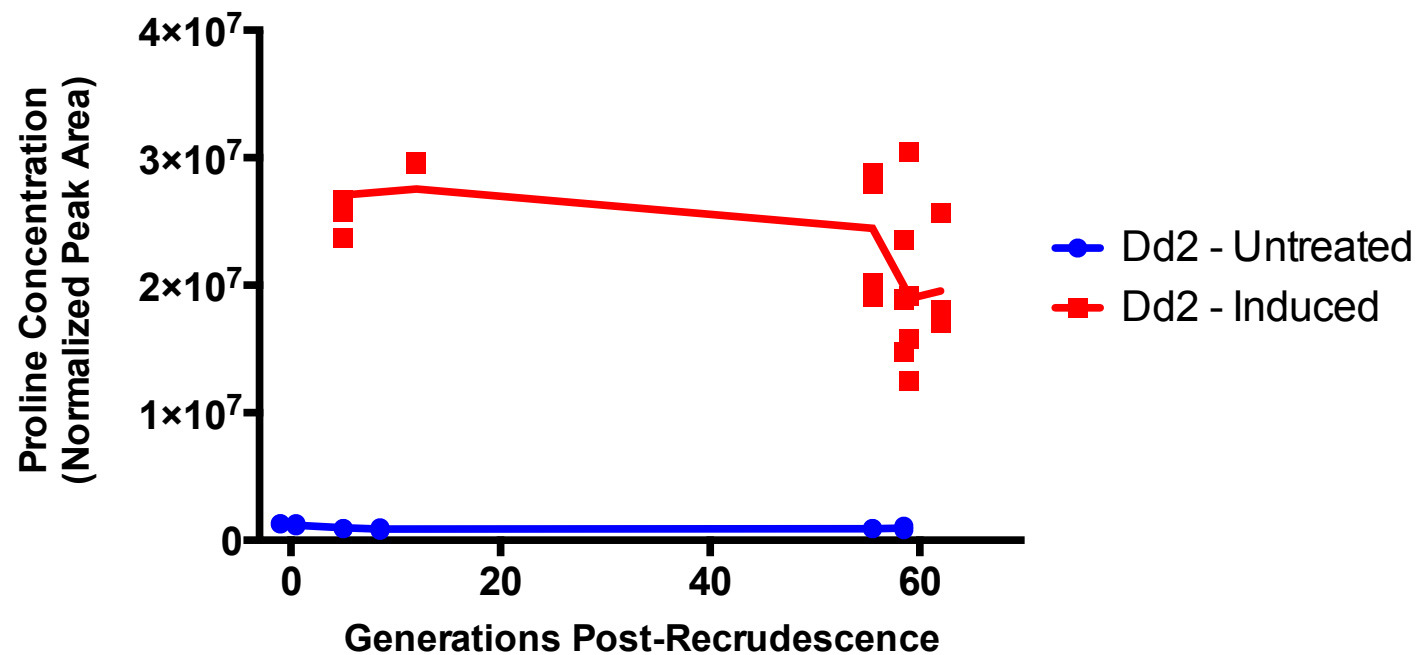

B

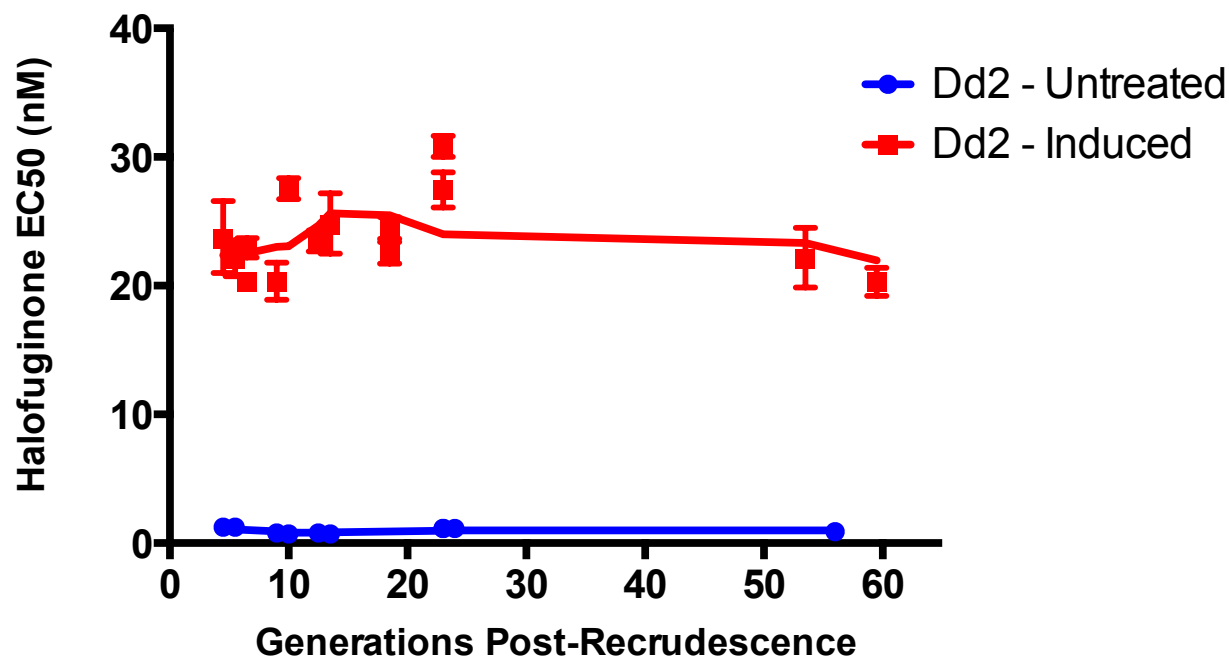

Supplement: Additional file 12: Figure S6. — Early dynamics of proline and halofuginone resistance in Dd2-induced parasites. After eight generations of exposure to 4× EC50 of halofuginone (2.8 nM), Dd2-induced parasites recrudesced. (a) As early as five generations post-recrudescence, Dd2-induced parasites had elevated intracellular proline levels that remained constantly elevated for an additional 55 generations. Proline levels of saponin-released P. falciparum parasites were quantified from the normalized integrated peak intensity from LC-MS analysis. (b) Dd2 parasites five generations post-recrudescence had also reached their maximum halofuginone resistance (EC50s 25 to 30 nM). Fifty-five generations later, the halofuginone dose-response in Dd2-induced parasites was unchanged. Error bars in (b) denote 95% confidence interval. [file 13059_2014_511_MOESM12_ESM.pdf]

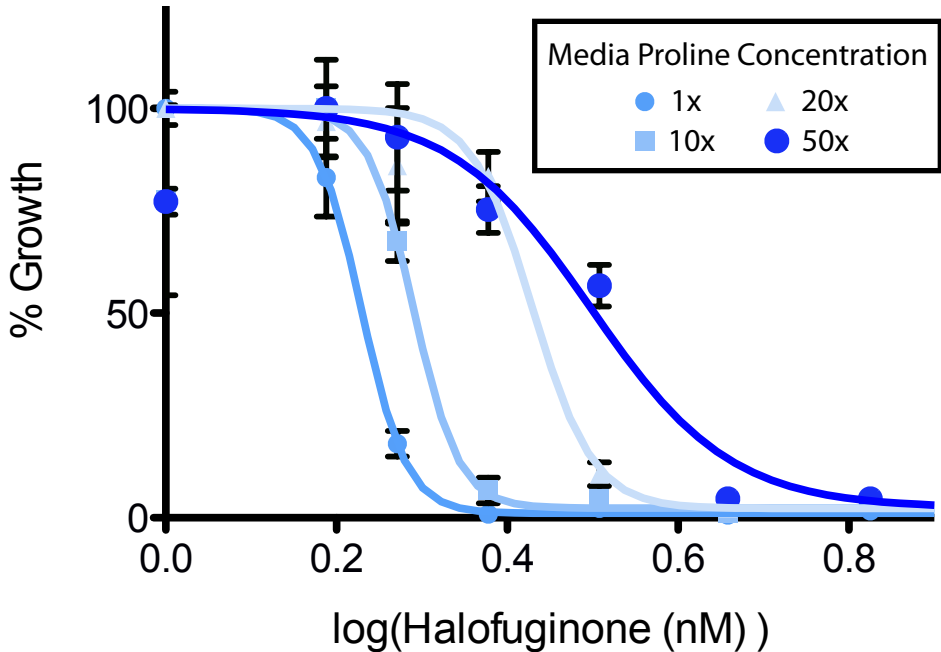

Supplement: Additional file 13: Figure S7. — Dose-dependent inhibition of P. falciparum growth is dependent on the concentration of free proline. SYBR Green dose response assays were performed with untreated (WT) Dd2 parasites that were put in media with four different proline concentrations at the start of the 72-h assay. The proline concentration of different in vitro culture medias is expressed relative to unmodified RPMI media. Error bars reflect standard deviations of triplicate experiments. [file 13059_2014_511_MOESM13_ESM.pdf]

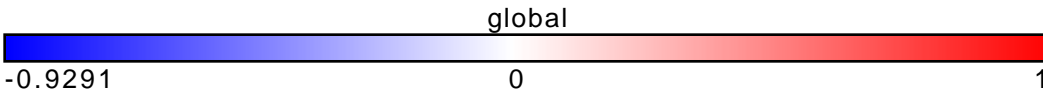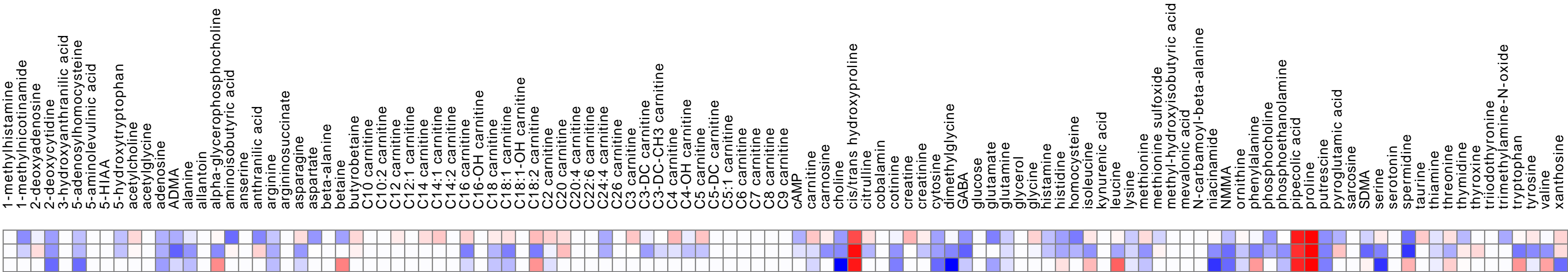

Id

Dd2 Induced

Dd2 Long Term Selected HFGR Lines

HB3 Induced

Supplement: Additional file 14: Figure S8. — Correlation of full metabolite profile with proline concentrations in halofuginone short-term induced and long-term selected parasites. Of the 115 polar metabolites measured, only pipercolic acid and cis/trans-hydroxyproline concentrations are highly correlated with cytosolic proline concentrations in short-term induced Dd2 parasites, short-term induced HB3 parasites, and long-term selected Dd2 lines HFGRI, II, and III. This heat map represents the correlation coefficients (r-values) between cytosolic concentrations of metabolites with that of proline in three collections of wild-type and modified parasites: short-term induced Dd2 parasites, short-term induced HB3 parasites, and long-term selected Dd2 HFGR lines. High correlation (r = 1) is represented by red while low correlation (r = -1) is represented by blue. [file 13059_2014_511_MOESM14_ESM.pdf]

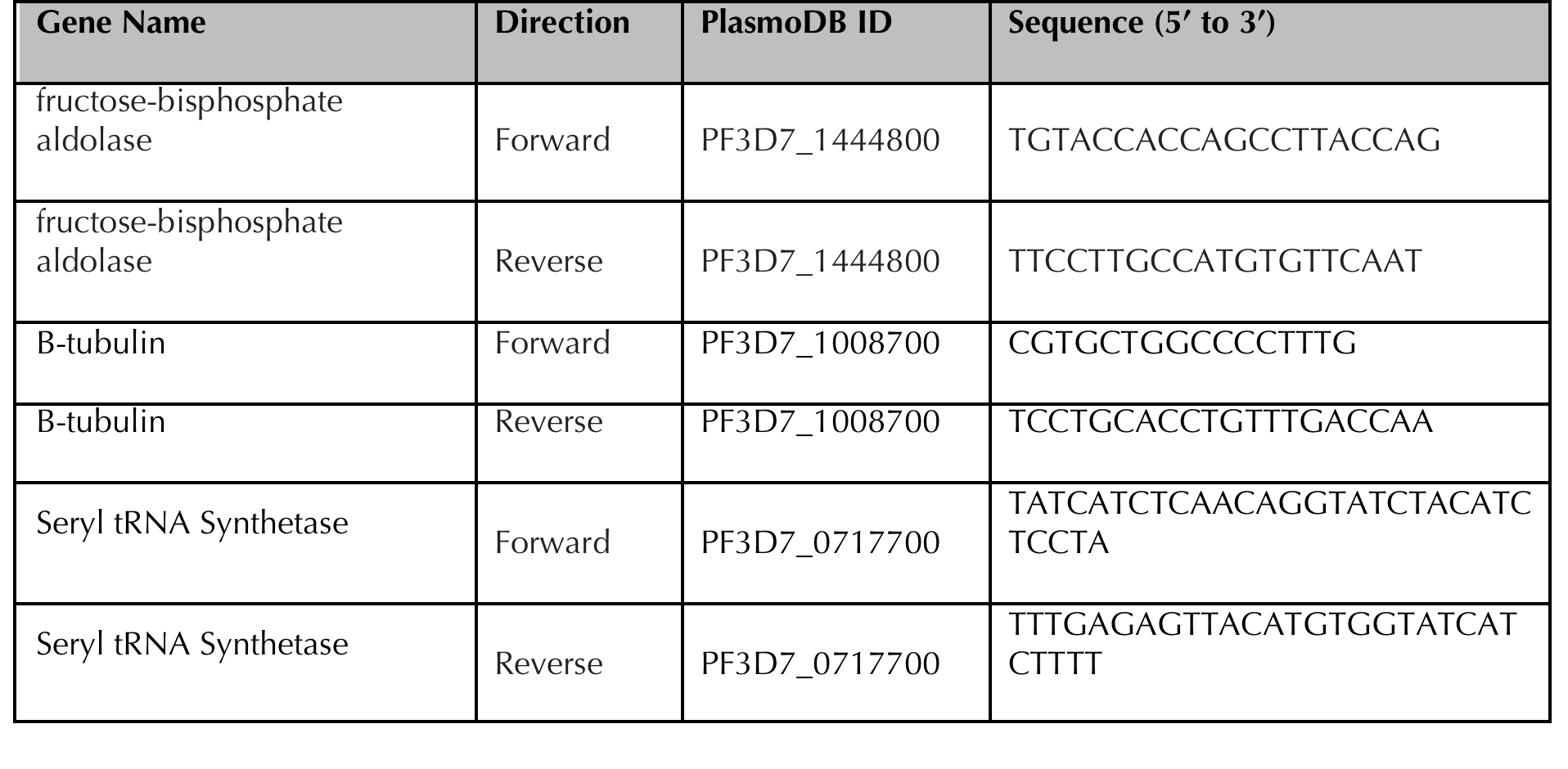

Supplement: Additional file 16: Table S7. — Quantitative PCR control primers for ∆∆Ct analysis. [file 13059_2014_511_MOESM16_ESM.doc]

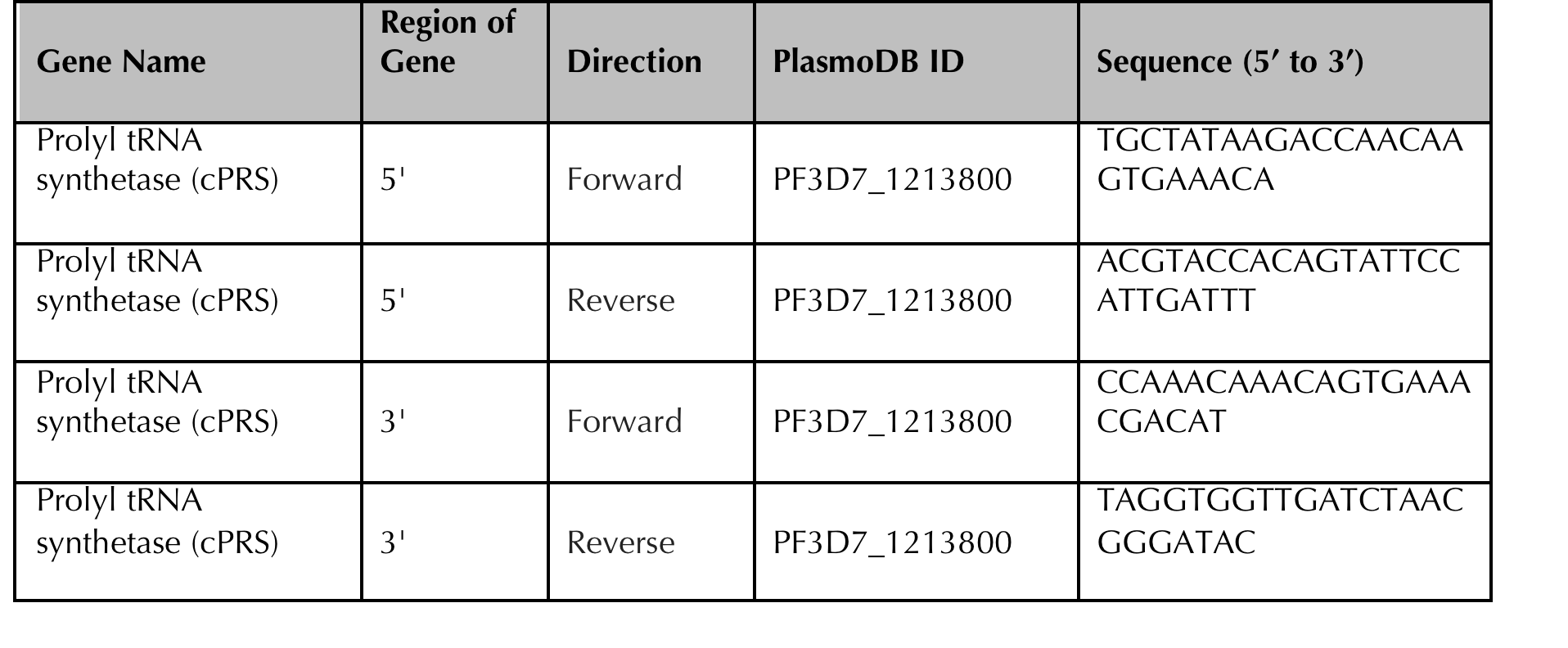

Supplement: Additional file 17: Table S8. — Quantitative PCR primers for the cPRS target locus analysis. [file 13059_2014_511_MOESM17_ESM.doc]
