# Supplementary material for: A genomic and evolutionary approach reveals non-genetic drug resistance in malaria
Source: Genome Biol. 2014 Nov 14;15(11):511. doi: 10.1186/s13059-014-0511-2 (PMC4272547; doi:10.1186/s13059-014-0511-2)
Supplement: Additional file 4: Table S3. — Filtering scheme for metagenomic sequence analysis of HFGRII and HFGRIII. [file 13059_2014_511_MOESM4_ESM.doc]

Filter Number of sites

------------------------------------------ -----------------

Total GATK putative mutations 127018

Average coverage > 10x 51082

No coverage in 3 indices 3135

Max in-population frequency > 0.10 1721

Average out-of-population frequency < 0.01 70990

Autocorrelation >= 0 73

Number of supporting reads > 10 14

Remaining sites 3
