# Supplementary material for: A genomic and evolutionary approach reveals non-genetic drug resistance in malaria
Source: Genome Biol. 2014 Nov 14;15(11):511. doi: 10.1186/s13059-014-0511-2 (PMC4272547; doi:10.1186/s13059-014-0511-2)
Supplement: Additional file 9: Figure S3. — Induced Dd2 parasites show no evidence of genetic modifications at the cPRS locus. (a) Sanger sequencing across the cPRS allele revealed that induced and untreated Dd2 strains have WT cPRS alleles. The codon where the HFGRI (T14445A) and HFGRII (C1444T) mutations occurred is highlighted in yellow. (b) Expression of cPRS in synchronous early schizonts is invariant between untreated and induced Dd2 parasite lines. [file 13059_2014_511_MOESM9_ESM.pdf]

A

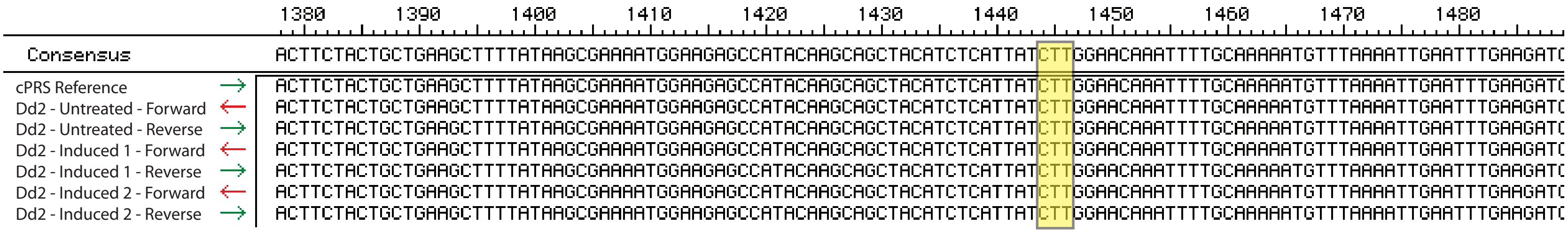

1380 1390 1400 1410 1420 1430 1440 1450 1460 1470 1480
Consensus ACTTCTACTGCTGAAGCTTTTATAAGCGAAAATGGAAGAGCCATACAAGCAGCTACATCTCATTATCTTGGAACAAATTTTGCAAAAATGTTTAAAATTGAATTTGAAGATC
cPRS Reference ACTTCTACTGCTGAAGCTTTTATAAGCGAAAATGGAAGAGCCATACAAGCAGCTACATCTCATTATCTTGGAACAAATTTTGCAAAAATGTTTAAAATTGAATTTGAAGATC
Dd2 - Untreated - Forward ACTTCTACTGCTGAAGCTTTTATAAGCGAAAATGGAAGAGCCATACAAGCAGCTACATCTCATTATCTTGGAACAAATTTTGCAAAAATGTTTAAAATTGAATTTGAAGATC
Dd2 - Untreated - Reverse ACTTCTACTGCTGAAGCTTTTATAAGCGAAAATGGAAGAGCCATACAAGCAGCTACATCTCATTATCTTGGAACAAATTTTGCAAAAATGTTTAAAATTGAATTTGAAGATC
Dd2 - Induced 1 - Forward ACTTCTACTGCTGAAGCTTTTATAAGCGAAAATGGAAGAGCCATACAAGCAGCTACATCTCATTATCTTGGAACAAATTTTGCAAAAATGTTTAAAATTGAATTTGAAGATC
Dd2 - Induced 1 - Reverse ACTTCTACTGCTGAAGCTTTTATAAGCGAAAATGGAAGAGCCATACAAGCAGCTACATCTCATTATCTTGGAACAAATTTTGCAAAAATGTTTAAAATTGAATTTGAAGATC
Dd2 - Induced 2 - Forward ACTTCTACTGCTGAAGCTTTTATAAGCGAAAATGGAAGAGCCATACAAGCAGCTACATCTCATTATCTTGGAACAAATTTTGCAAAAATGTTTAAAATTGAATTTGAAGATC
Dd2 - Induced 2 - Reverse ACTTCTACTGCTGAAGCTTTTATAAGCGAAAATGGAAGAGCCATACAAGCAGCTACATCTCATTATCTTGGAACAAATTTTGCAAAAATGTTTAAAATTGAATTTGAAGATC

B

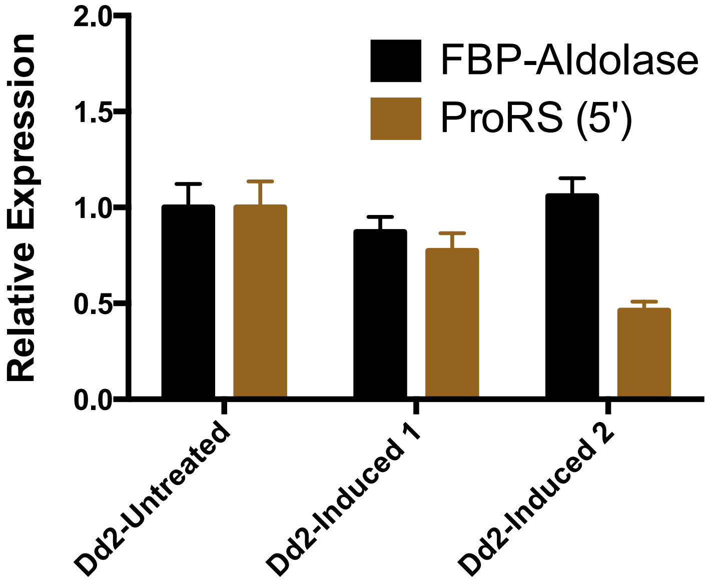

Relative Expression
2.0
1.5
1.0
0.5
0.0
FBP-Aldolase
ProRS (5')
Dd2-Untreated
Dd2-Induced 1
Dd2-Induced 2
