# Supplementary material for: A genomic and evolutionary approach reveals non-genetic drug resistance in malaria
Source: Genome Biol. 2014 Nov 14;15(11):511. doi: 10.1186/s13059-014-0511-2 (PMC4272547; doi:10.1186/s13059-014-0511-2)
Supplement: Additional file 10: Table S6. — SNPs called between whole-genome sequencing of Dd2 untreated and Dd2 Induced 1 and Dd2 Induced 2 lines. [file 13059_2014_511_MOESM10_ESM.doc]

Five genes were identified with non-synonymous SNPS in coding regions of the P. falciparum genome that differed between the parental Dd2 and Dd2-Induced 1 and 2 strains. Upon Sanger re-sequencing, all except the SNP in PF3D7_072780, were attributed to sequencing/alignment/SNP calling errors often caused by the challenge of calling deletions in WGS data.
