# Supplementary material for: A genomic and evolutionary approach reveals non-genetic drug resistance in malaria
Source: Genome Biol. 2014 Nov 14;15(11):511. doi: 10.1186/s13059-014-0511-2 (PMC4272547; doi:10.1186/s13059-014-0511-2)
Supplement: Additional file 15: Figure S9. — Expression analysis of Dd2-induced parasites. (a) qPCR-based expression analysis of the clag family of transport-involved genes demonstrates that their differential expression is not involved in Dd2 induced halofuginone resistance. Relative expression was calculated with the ddCt method using SerRS as control and in reference to a stage-matched untreated Dd2 parasite. FBP-Aldolase and ProRS (5′) are provided as additional controls. Results represent eight biological replicate measurements (each data point) each determined by technical quadruplicate. (b) Spearman correlation was used to directly identify the stage of the three Dd2 RNA-Seq libraries assembled. (c) Log-transformed fold change of replicate Dd2 inductions (Dd2-Induced 1 and Dd2-Induced 2) (y-axis) was graphed with the log-transformed fold change from a 3D7 RNA-Seq control time course (x-axis) between corresponding stages (40 vs 36 hours post-infection ). By identifying outliers from this linear fitting, (d) we determined genes that are differential expressed between Dd2 induced and untreated parasites independent of stage-specific expression. [file 13059_2014_511_MOESM15_ESM.pdf]

A

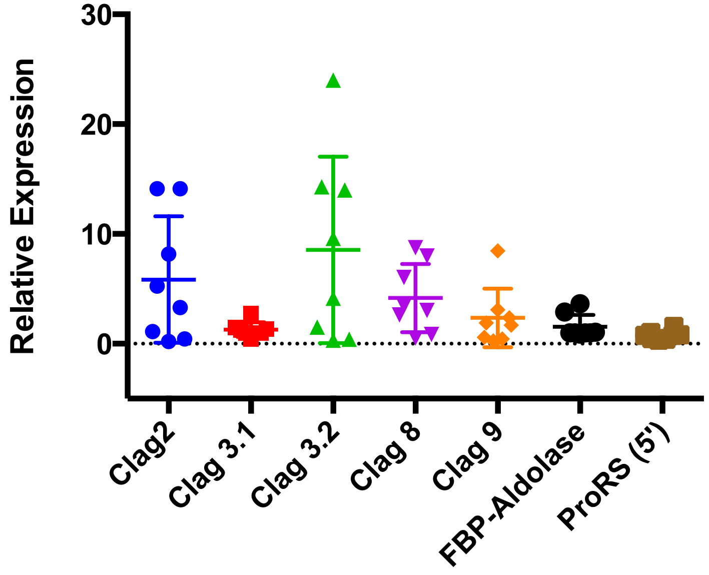

B

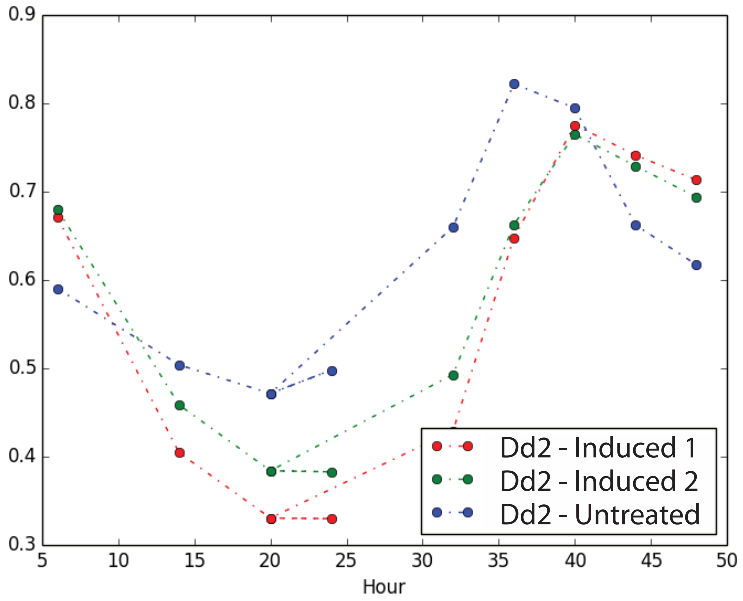

C

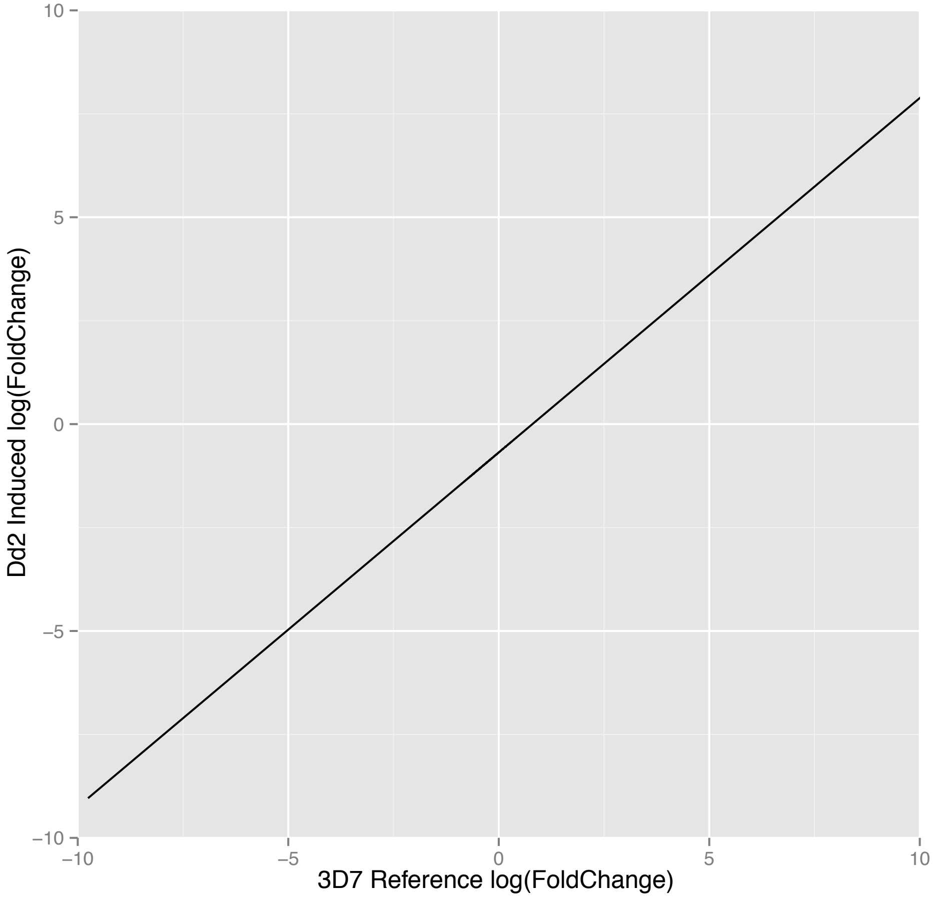

D

PlasmoDB ID	Gene Description	3D7 Life Cycle Control	Dd2 Induction 1	Dd2 Induction 2	Dd2 Induction - Average
PF3D7_0310600.2	SAC3/GNAP family-related protein, putative	11.81	-9.19	-8.55	-8.87
PF3D7_0318700	trophozoite stage antigen	4.95	13.16	14.83	14.00
PF3D7_0723200	conserved Plasmodium protein, unknown function	0.96	11.57	11.93	11.75
PF3D7_1209500.1	cGMP-specific phosphodiesterase (PDE1)	1.98	13.27	13.64	13.46
PF3D7_1209500.2	cGMP-specific phosphodiesterase (PDE1)	10.90	0.61	0.95	0.78
PF3D7_1233200.1	conserved Plasmodium protein, unknown function	1.22	11.26	12.92	12.09
PF3D7_1419800.1	glutathione reductase (GR)	-9.76	-1.51	-0.29	-0.90
