# Supplementary material for: A genomic and evolutionary approach reveals non-genetic drug resistance in malaria
Source: Genome Biol. 2014 Nov 14;15(11):511. doi: 10.1186/s13059-014-0511-2 (PMC4272547; doi:10.1186/s13059-014-0511-2)
Supplement: Additional file 18: Table S9. — Quantitative PCR primers for the clag gene family expression analysis. [file 13059_2014_511_MOESM18_ESM.doc]

| **Gene Name** | **Direction** | **PlasmoDB ID** | **Sequence (5’ to 3’)** |
| --- | --- | --- | --- |
| Clag 2 | Forward | PF3D7_0220800 | CTCTTACTACTTATTATCTATCTCTCA |
| Clag 2 | Reverse | PF3D7_0220800 | CCAGGCGTAGGTCCTTTAC |
| Clag 3.1 | Forward | PF3D7_0302500 | ACCCATAACTACATATTTTCTAGTAATG |
| Clag 3.1 | Reverse | PF3D7_0302500 | TCTGAACTAGGAGGCCAACC |
| Clag 3.2 | Forward | PF3D7_0302200 | ACCCATAACTACATATTTTCTAGTAATG |
| Clag 3.2 | Reverse | PF3D7_0302200 | TTCAGCAGCAAGTCCGTGA |
| Clag 8 | Forward | PF3D7_0831600 | GTTACTACAACATTCCTGATTCAG |
| Clag 8 | Reverse | PF3D7_0831600 | AATGAAAATATAAAAATGCTGGGGGAT |
| Clag 9 | Forward | PF3D7_0935800 | TACCATTAGTGTTTTATACACTTAAGG |
| Clag 9 | Reverse | PF3D7_0935800 | CCAAAATATGGCCAAGTACTTGC |
