# Supplementary material for: A genomic and evolutionary approach reveals non-genetic drug resistance in malaria
Source: Genome Biol. 2014 Nov 14;15(11):511. doi: 10.1186/s13059-014-0511-2 (PMC4272547; doi:10.1186/s13059-014-0511-2)
Supplement: Supplementary file 9 [file 13059_2014_511_MOESM5_ESM.doc]

MetaPop	Autocorr	Dd2Ref - # CHROM	Dd2Ref - POS	3D7Ref - # CHROM	3D7Ref - POS	Gene ID	REF	ALT	1-010-2	1-030-2	1-060-2	1-100-1	1-300-2	1-010-3	1-030-1	1-060-1	1-060-2	1-100-1	1-300-2	1-300-3	1-010-22	1-030-23	1-060-16	1-100-16	1-300-16	1-010-27	1-030-18	1-060-19	1-060-110	1-300-111	1-300-212	1-300-815
HPGR3I	0.783862785	12	1635603	10	1636896	Intergenic	G	T	0.02180952	0.00806452	0	0.00869565	0	0	0.01449275	0.00892857	0	0	0.025	0.22222222	1.00000001	0.99999998	0	0.99999998	0	0	2.00000005	0.99999993	0	0	1	23.9999997
HPGR3	0.11290392	14	590128	12	590901	PF3D7_1213...	G	A	0	0	0.39628906	0.946	0.22608586	0	0	0	0	0.00400128	0	0	0	0	141	101	28.9999999	0	0	0	0	0.99999998	0	0
HPGR3	0.89480952	16	97418	14	97432	PF3D7_1402...	A	T	0	0.0031157	0.0050587	0.0944827	0.0112091	0	0.0172433	0.0126582	0	0	0	0.0268157	0	1.00000002	0.99999999	1.00000001	21	0	0.99999998	1.00000001	0	0	0	1.99999998
